# Neural stem cell quiescence and stemness are molecularly distinct outputs of the Notch3 signalling cascade in the vertebrate adult brain

**DOI:** 10.1242/dev.161034

**Published:** 2018-05-15

**Authors:** Emmanuel Than-Trong, Sara Ortica-Gatti, Sébastien Mella, Chirag Nepal, Alessandro Alunni, Laure Bally-Cuif

**Affiliations:** 1 Institut Pasteur, Unit Zebrafish Neurogenetics, Department of Developmental & Stem Cell Biology, 25 rue du Dr Roux, 75015 Paris, France; 2 CNRS, UMR3738, 25 rue du Dr Roux, 75015 Paris, France; 3 Institut Pasteur, Unit Stem Cells and Development, Department of Developmental & Stem Cell Biology, 25 rue du Dr Roux, 75015 Paris, France; 4 Biotech Research and Innovation Centre, University of Copenhagen, 2200 Copenhagen, Denmark

**Keywords:** Notch3, Hey1, Quiescence, Stemness, Neural stem cell, Pallium

## Abstract

Neural stem cells (NSCs) in the adult vertebrate brain are found in a quiescent state and can preserve long-lasting progenitor potential (stemness). Whether and how these two properties are linked, and to what extent they can be independently controlled by NSC maintenance pathways, is unresolved. We have previously identified Notch3 signalling as a major quiescence-promoting pathway in adult NSCs of the zebrafish pallium. We now show that Notch3 also controls NSC stemness. Using parallel transcriptomic characterizations of *notch3* mutant NSCs and adult NSC physiological states, we demonstrate that a set of potentially direct Notch3 target genes distinguishes quiescence and stemness control. As a proof of principle, we focus on one ‘stemness’ target, encoding the bHLH transcription factor Hey1, that has not yet been analysed in adult NSCs. We show that abrogation of Hey1 function in adult pallial NSCs *in vivo*, including quiescent NSCs, leads to their differentiation without affecting their proliferation state. These results demonstrate that quiescence and stemness are molecularly distinct outputs of Notch3 signalling, and identify Hey1 as a major Notch3 effector controlling NSC stemness in the vertebrate adult brain.

## Abstract (precis)

**Highlighted Article:** Notch3 signalling selectively controls neural stem cell stemness through the activity of the Hey1 transcription factor, independently of its molecular regulation of neural stem cell quiescence.

## INTRODUCTION

Neural stem cells (NSCs) are astroglial cells sitting at the top of a cell hierarchy leading to the generation of new neurons and glial cells in the adult vertebrate brain. They are physiologically crucial components of brain physiology, but the cell-intrinsic and population mechanisms that account for their life-long preservation are incompletely understood. Essential parameters of NSC maintenance include stemness and quiescence, although to what extent both processes are linked is a matter of debate. Stemness, or long-lasting progenitor properties, is a functional parameter that is difficult to rigorously assess in the brain. Through cell tracing, NSC stemness has been associated with the expression of ‘upstream’ progenitor markers (such as the transcription factor Sox2) (Graham et al., 2003; Suh et al., 2007; Codega et al., 2014; Favaro et al., 2009), and with the capacity to divide, self-renew and generate progeny oriented towards the neuronal lineage. Quiescence is defined as the non-dividing state of cells harbouring progenitor potential. Often corresponding to the G0 state, it is thus characterized by the lack of expression of proliferation markers such as proliferating cell nuclear antigen (PCNA) or mini-chromosome maintenance (MCM) proteins (Valcourt et al., 2012) and will be referred to as such, i.e. a non-proliferating but proliferation-competent cell state, in this paper. Quiescence is profound in adult NSCs (Temple, 2001; Fuentealba et al., 2012; Ming et al., 2011; Wang et al., 2011). In several systems, it is interpreted to favour the preservation of stem cell properties by decreasing the risk of accumulating mutations during DNA replication, and to permit energy sparing and limit the production of reactive oxygen species (Nijnik et al., 2007; Rossi et al., 2007; Valcourt et al., 2012; Cavallucci et al., 2016). In addition, and although the mechanisms are less understood, quiescence exit may be linked with NSCs entering an alternative state of more-frequent divisions, or may participate in a process that ‘counts’ division events, ultimately leading to NSC exhaustion (Encinas et al., 2011; Encinas and Sierra, 2012; Urbán et al., 2016). Overall, understanding how stemness and quiescence are encoded, and their potential links, is of fundamental interest and extends beyond the NSC field.

At the molecular level, a number of intrinsic and extrinsic factors have been identified to control adult NSC stemness or quiescence, or both. Abrogation of SOX2 (Favaro et al., 2009) or TLX (Liu et al., 2008) function, or decreased ROS levels (Le Belle et al., 2011) in NSCs of the adult mouse brain, lead to loss of NSC function, in the absence of reported proliferation increase. These factors may selectively promote stemness, whether during the quiescence phase or upon NSC division (self-renewal). In contrast, decreased levels of the transcription factors NFIX and HUWE1 (Martynoga et al., 2013; Andersen et al., 2014; Urbán et al., 2016), of BMP signalling (Bonaguidi et al., 2008; Mira et al., 2010; Sun et al., 2011; Martynoga et al., 2013), and of insulin signalling and its downstream targets (FOXO proteins) (Paik et al., 2009; Renault et al., 2009; Webb et al., 2013), appear to primarily impact NSC quiescence, leading to excessive NSC proliferation. However, the primary targets of several pathways remain unresolved, among which is Notch signalling, a crucially relevant regulator of adult NSC maintenance. Notch signalling converges onto the transcription factor RBPj, which is bound by the Notch intracellular domains after its translocation to the nucleus. Invalidating RBPj triggers a boost of proliferation within the SOX2-positive population of the adult mouse subgranular zone of the hippocampus (SGZ), which is accompanied by stemness loss and NSC depletion (Ehm et al., 2010). Parallel data were obtained in the sub-ependymal zone of the lateral ventricle (SEZ), although they were more difficult to interpret as NSC and proliferation markers were not combined (Imayoshi et al., 2010). Finally, inactivation of the Notch ligands Jagged1 or Delta-like 1 in NSC-contacting cell populations, such as blood vessels or transit amplifying progenitors, respectively, triggers NSC activation (Ottone et al., 2014; Kawaguchi et al., 2013). Although these studies pointed to a primary role of Notch signalling in the control of NSC quiescence, these phenotypes were tracked at the population level and it could not be concluded whether Notch also directly controls NSC stemness in quiescent NSCs. In addition, Notch effector genes remain incompletely characterized.

In the zebrafish adult dorsal telencephalon (pallium), which hosts the homologous domains to the rodent SEZ and SGZ, Notch signalling function could be partially resolved through detailed expression analyses of Notch ligands and their selective abrogation. Adult NSCs in this domain are radial glial cells (RGs), which exhibit similar properties to their rodent counterparts: they are self-renewing, multipotent, strongly quiescent (with no more than 5-10% of NSCs in cycle – referred to as ‘activated’ – at a given time point) and express the transcription factor Her4, which is orthologous to mammalian HES5 (Adolf et al., 2006; Grandel et al., 2006; Chapouton et al., 2010). We have previously shown that quiescent RGs (qRGs) express the Notch3 receptor, whereas activated RGs (aRGs) express both Notch3 and Notch1, and that the selective abrogation of Notch3 and Notch1 affects quiescence and self-renewal, respectively (Rothenaigner et al., 2011; Alunni et al., 2013). In the absence of Notch3 [in the null mutant *notch3^fh332^* or upon *notch3* morpholino (*notch3*MO) electroporation into adult NSCs *in vivo*], the proportion of activated RGs is significantly increased [1.4-fold in 7-days post-fertilization (7 dpf) *notch3^fh332^* larvae, threefold in *notch3*MO adults]. The control of adult NSC quiescence by NOTCH3 was recently confirmed in mouse (Kawai et al., 2017). In contrast, in the absence of Notch1, 79% of activated RGs chose neuronal differentiation instead of self-renewal (Alunni et al., 2013). This function is also paralleled by mouse NOTCH1 in the adult SEZ and SGZ (Ables et al., 2010; Basak et al., 2012). The expression of Notch3 in aRGs, however, suggests a function that is additional to the control of quiescence, and the state of qRGs that do not reactivate upon Notch3 abrogation remains to be addressed.

To address these issues, we have traced pallial neural progenitor cell fate upon *notch3* invalidation, revealing an unexpected function for Notch3 in stemness in addition to quiescence control. To understand the molecular support for this function, we designed a double-transcription profiling approach to uncover Notch3 targets in pallial RGs and to position them relative to RG states. Our results suggest that Notch3 signalling promotes both quiescence and stemness through, at least in part, distinct downstream mediators. Further validation of one of these targets, the bHLH transcription factor Hey1, in adult NSCs *in vivo*, indeed demonstrates its selective involvement in stemness but not in quiescence control.

## RESULTS

### Notch3 controls pallial neural progenitor stemness

**Fig. 1.**
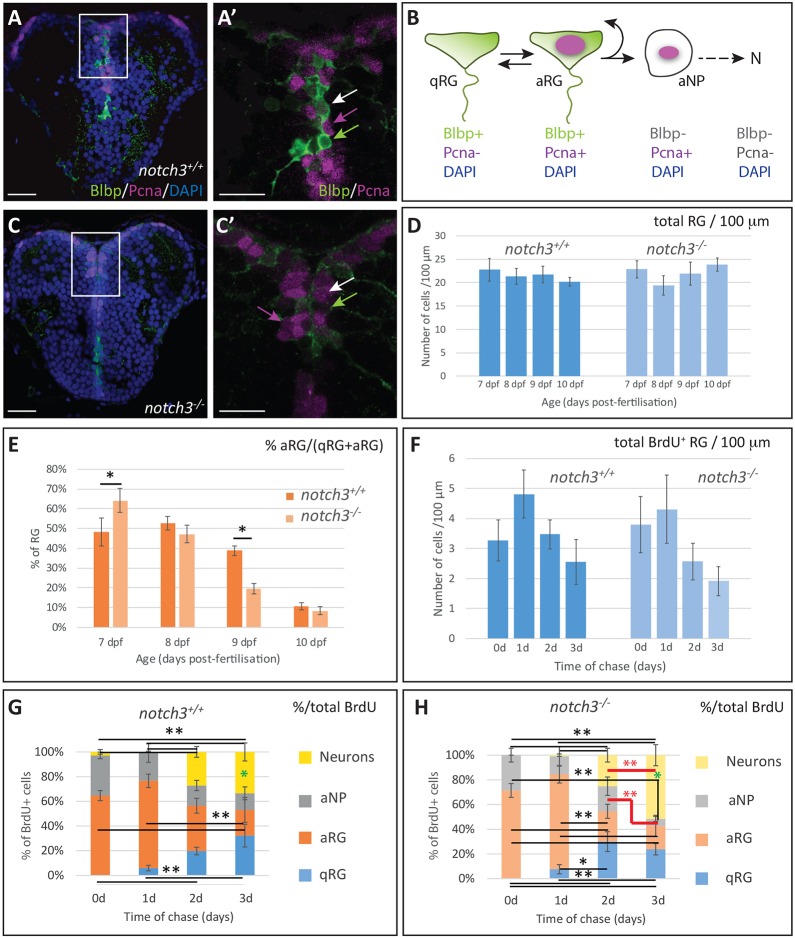
**Notch3 controls radial glia quiescence and stemness.** (A-B) Detection of the three progenitor cell types of the pallial VZ in a wild-type 7 dpf larva. (C) Progenitors of the pallial VZ in a 7 dpf *notch3^−/−^* larva. (A,C) Double immunocytochemistry for the RG marker BLBP (green) and the proliferation marker PCNA (magenta) on a telencephalic cross-section (counterstained with DAPI). (A′,C′) High magnification of the areas boxed in A,C. qRG, green arrow; aRG, white arrow; aNPs, magenta arrow. (B) Schematic representation of the main neurogenic cascade in the post-embryonic pallium, with diagnostic markers. At least some RGs transit between the qRG and aRG states (Chapouton et al., 2010). N, neurons. (D) Total number of RGs (qRGs+aRGs) counted per 100 µm of VZ on cross-sections at mid-pallial levels. There is no significant difference between stages and between genotypes within the period considered. (E) Proportion of aRGs within the total RG population between 7 dpf and 10 dpf compared in wild-type and *notch3^−/−^* sibling larvae. *P<0.05 after Holm's correction, otherwise non-significant. (F) Total number of BrdU-positive RGs (qRGs+aRGs) counted per 100 µm of VZ on cross-sections at mid-pallial levels between 7 dpf (t0, no chase) and 10 dpf (3 days of chase), compared in wild-type and *notch3^−/−^* sibling larvae. (G,H) Proportion of the different neural cell types (qRGs, aRGs, aNPs, neurons) within the BrdU-positive population following BrdU pulse application at 7 dpf (t0, no chase) and after 1, 2 or 3 days of chase (i.e. with analyses at 8, 9 and 10 dpf, respectively), compared in wild-type (G) and *notch3^−/−^* (H) sibling larvae. Black lines and asterisks: statistics with Holm's correction for multiple comparisons. *P<0.05, **P<0.01. Red lines and asterisks: LSD test for comparisons between 2 and 3 days of chase. The proportion of aNPs decreases significantly and the proportion of neurons increases significantly, in *notch3^−/−^* mutants only (*P*=0.007 and *P*=0.002, respectively). Green asterisks: LSD test for comparisons between wild-type and *notch3^−/−^* at 3 days of chase. The proportion of neurons is significantly increased in *notch3^−/−^* mutants versus wild type (*P*=0.02). Scale bars: 10 µm in A,C; 20 µm in A′,C′. (D-F) *n*=6-11 telencephali per condition.

We have previously observed that RG quiescence normally initiates around 7 dpf in the larval pallium (90% of pallial RGs proliferating at 5 dpf, but only 70% at 7 dpf), whereas most pallial RGs remain activated in 7 dpf homozygous *notch3^fh332^* mutants (hereafter referred to as *notch3^−/^*^−^) (Alunni et al., 2013). The consequences of *notch3* function abrogation past 7 dpf were, however, not analysed. To assess the immediate fate of pallial RGs in *notch3^−/−^* mutants, we first analysed cell identities over time in the pallial germinal zone during the period preceding larval lethality (around 10-15 dpf). RGs were identified by their expression of fatty acid-binding protein 7a (Fabp7a, also called brain lipid-binding protein – Blbp), and the proliferating progenitor population by its expression of proliferating cell nuclear antigen (Pcna) or mini-chromosome maintenance (Mcm) proteins. These markers, as in the adult, identify the three ventricular progenitor cell states/types in the larval pallium: quiescent RGs (qRGs) (BLBP^+^, PCNA/MCM^−^), activated RGs (aRGs) (Blbp^+^, Pcna/Mcm^+^) and proliferating non-RG neural progenitors (aNPs) (BLBP^−^, PCNA/MCM^+^) (Fig. 1A,B) (Alunni et al., 2013). In wild-type larvae, we observed that the total number of RGs (qRGs+aRGs) (Fig. 1A,D), the total number of progenitors (qRGs+aRGs+aNPs) (Fig. S1J), and the proportion of glial (qRGs+aRGs) and non-glial progenitors within the progenitor population (Fig. S1K) were maintained roughly constant between 7 and 10 dpf. However, the proportion of aRGs among the whole RG population progressively decreased, from 48% at 7 dpf to 11% at 10 dpf (Fig. 1E, Fig. S1I,K), reflecting the progression of quiescence instatement in pallial RGs. In *notch3^−/−^* larvae, however, the proportion of aRGs within the RG population was initially (at 7 dpf) increased, reflecting the previously reported Notch3 function in promoting RG quiescence, but, at 9 dpf, exhibited a decrease much stronger than in wild type (Fig. 1C,E). To determine whether cell death played a role in this phenotype, we analysed expression of phospho-caspase3, but found no evidence for RG death at any stage in wild-type or *notch3^−/−^* larvae between 7 and 10 dpf (Fig. S1L). In addition, we found that the total number of RGs in *notch3^−/−^* was constant over this time period and similar to that in wild-type larvae (Fig. 1D). Together, these observations suggest anticipated RG cell cycle exit in mutants.

To interpret the bias in RG fate in *notch3^−/−^* mutants, we used a BrdU pulse-chase analysis to trace aRGs. A 5 h BrdU pulse was applied at 7 dpf, and the identity of BrdU-positive cells was assessed until 10 dpf (Fig. 1G,H; Fig. S1A-H′,M,N). The proportion of aRGs is higher than aNPs at this stage in the progenitor population (67% compared with 33% in wild-type larvae, 72% compared with 28% in *notch3^−/−^* mutants), which is also reflected in the identity of BrdU-positive cells immediately after the pulse (Fig. 1G,H). Thus, this experimental scheme mostly traces aRG fate. BrdU-positive cells negative for RGs and/or proliferation markers were scored as neurons, in agreement with the sole generation of neurons as a non-progenitor population from the pallial VZ at these stages (Dirian et al., 2014). Between 7 and 10 dpf (0 to 3 days of chase), the number of BrdU-positive RGs and aNPs decreased in wild-type and *notch3^−/−^* larvae, whereas the number of neurons increased, reflecting neuronal generation from RGs (Fig. 1F, Fig. S1M,N). However, we found that, between 2 and 3 days of chase (9 and 10 dpf), the proportion of neurons increased significantly in *notch3^−/−^* mutants, with a concomitantly significant decrease in the proportion of aNPs, although these values were not significantly changed in wild-type siblings (Fig. 1G,H). Likewise, the proportion of neurons is significantly higher in *notch3^−/−^* larvae when comparing wild type and mutants after 3 days of chase (10 dpf). These observations suggest that *notch3^−/−^* RGs prematurely commit to neurogenesis at 9-10 dpf. Together, the findings above indicate that, in addition to promoting RG quiescence, Notch3 is necessary to maintain the RG progenitor state. Combined with our previous data (Alunni et al., 2013), these results suggest a dual function for Notch3 in post-embryonic RGs: the maintenance of both quiescence and of the progenitor state. To dissect the mechanisms underlying these activities, we designed profiling experiments and functional assays aiming to identify and categorize Notch3 targets (Fig. S2).

### Identification of Notch3 transcriptional targets in radial glia

**Fig. 2.**
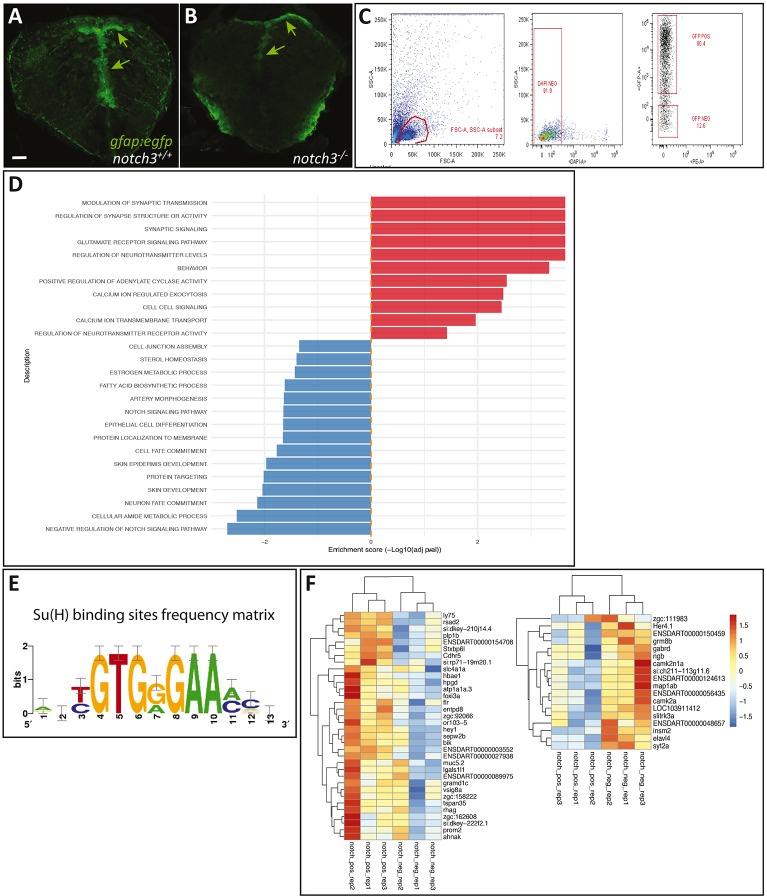
**RNAseq identification of Notch3-dependent genes in 7 dpf radial glia.** (A,B) Radial glia cells (RGs) (*gfap:gfp*, green, arrows) observed on cross-sections of the 7 dpf telencephalon in *notch3^+/+^* and *notch3^−/−^* sibling larvae, used for FACS sorting. (C) Representative FACS dot plots showing the gating strategy. FSC/SSC plot and selected cells, which are then gated for DAPI negativity (middle panel) and for GFP expression (right panel). (D) List of significantly enriched GO terms (ordered by enrichment score) within the list of DEGs in 7 dpf RGs between *notch3^−/−^* and wild-type sibling larvae (red, enriched in mutants; blue, enriched in wild type) (see also Table S4). (E) Position-weight matrix for RBPJ/Su(H)-binding sites used in the present study (graphical representation). (F) Heat maps of the genes down- or upregulated in *notch3^−/−^* compared with *notch3^+/+^* larvae and harbouring putative RBPJ-binding sites. Cutoff on display: log(fold change)>1. See also Tables S1 and S2. Scale bar: 20 µm.

To identify Notch3 molecular targets in NSCs, we compared the transcriptome of pallial RGs in *notch3^−/−^* mutants and wild-type siblings. The glial fibrillary acidic protein gene (*Gfap*) is, like *blbp*, selectively expressed in RGs (Alunni et al., 2013). Hence, to isolate RGs, the *notch3^fh332^* line was crossed into the *Gfap:egfp* transgenic background (Bernardos and Raymond, 2006) and eGFP-positive cells were sorted from genotyped 7 dpf larval heads (Fig. 2A-C). Overall, three independent batches of 15 larval heads for each genotype were sorted using FACS and processed (see Materials and Methods). After correcting evident batch effects, PCA analysis on the 500 genes showing the highest variability confirmed that genotype was the most important discriminatory factor between biological replicates under these conditions (Fig. S3A). We recovered a total of 284 differentially expressed genes (adjusted *P*-value<0.05), including 197 downregulated and 87 upregulated genes, between *notch3^−/−^* and wild-type pallial RGs (Fig. S3B,C, Tables S1 and S2). GO term analyses highlighted biological pathways prominently mis-regulated in pallial RGs in the absence of Notch3 (Fig. 2D, Table S3). Genes related to neurotransmitter signalling and metabolism, synaptic transmission, calcium transport and cell-cell signalling were significantly upregulated in mutants (hence, corresponding to functions antagonized by Notch3 activity) (Fig. 2D). In contrast, genes involved in cell junction assembly and cell metabolism were downregulated, as well as pathways involved in cell fate commitment or determination (notably neuronal fate) and, expectedly, Notch signalling (Fig. 2D). These last gene sets (involved in cell fate commitment or determination) therefore appear to be positively dependent, directly or indirectly, on Notch3 signalling for their expression.

Canonical Notch signalling involves association of the Notch intracellular fragment (NICD) with the transcription factor RBPJ on DNA at consensus sites (Jarriault et al., 1995; Kopan et al., 2009). To determine which differentially regulated genes harbour a potential RBPJ-binding site – and in the absence of efficient antibodies against the zebrafish RBPJ protein – we used the matrix-scan tool of the RSAT suite with a position weight matrix based on sequences bound by Su(H) (the *Drosophila* orthologue of RBPJ) (Fig. 2E) to screen 2 kb upstream of the predicted transcription start sites of each recovered gene. We found that 36 downregulated genes in *notch3^−/−^* mutants and 17 upregulated genes, harboured, with 95% confidence, a predicted RBPJ-binding site in their upstream sequence (Fig. 2F, Table S4). These genes are potentially directly regulated by Notch3 in pallial RGs. RT-qPCR validation was successfully achieved for two targets (*hey1*, *plp1b*) from four tested genes (including also *prom2 *and* ly75*) (Fig. S3D). To obtain enough RNA material, whole heads instead of FACS-sorted RGs were used in the RT-qPCR validation, potentially buffering the effect of the *notch3* mutation for broadly expressed genes.

### Notch3 target genes in pallial radial glia distribute in subclasses highlighting quiescence or a stemness/progenitor state

**Fig. 3.**
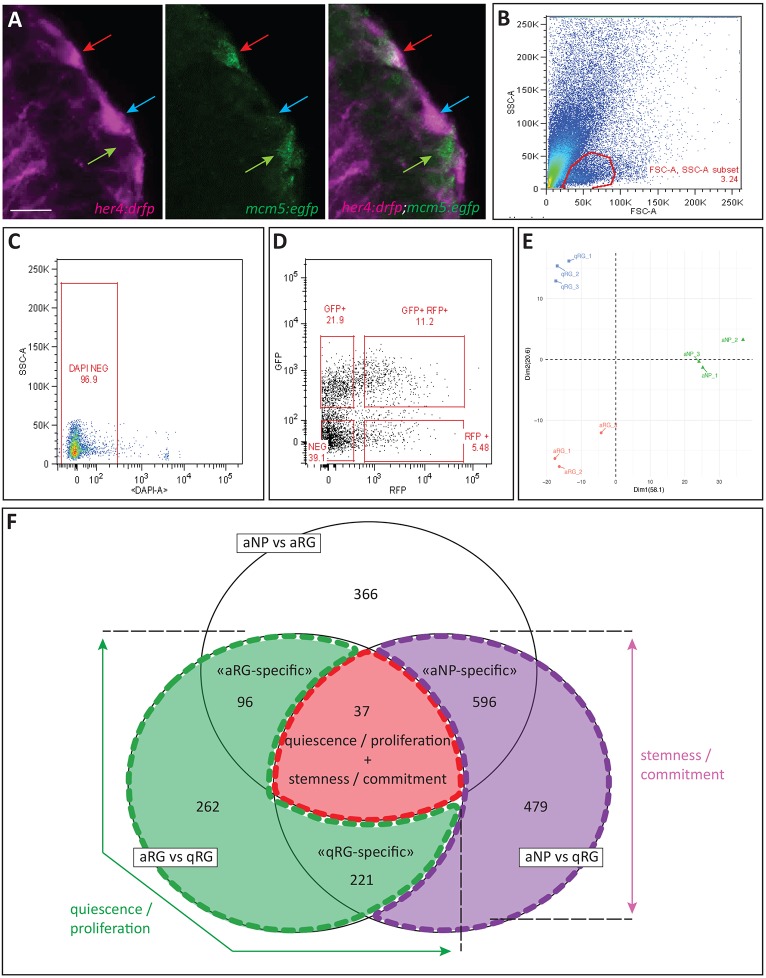
**RNAseq identification of RG quiescence and stemness markers.** (A) High-magnification view of a pallial VZ area in *her4:drfp;mcm5:egfp* double transgenic adult, highlighting the FACS-sorted progenitor types. Cross-section processed by immunocytochemistry for RFP (magenta) and GFP (green) (left panel, magenta channel; middle panel, green channel; right panel, merge). Coloured arrows indicate the different cell types (blue, qRGs; red, aRGs; green, aNPs) (as in E). Scale bar: 10 μm. (B-D) Representative FACS dot plots showing the gating strategy. FSC/SSC plot and selected cells (B,C), which are then gated on RFP and GFP intensities (D). (E) PCA analysis on the 500 showing the greatest variability across the different FACS-sorted biological replicates (blue, qRGs; red, aRGs; green, aNPs). (F) Venn diagram illustrating the position of recovered DEGs between the different cell state comparisons and their biological interpretation.

We next aimed to position these potentially direct Notch3 target genes relative to Notch3-related NSC properties, notably quiescence and stemness. For this, we designed a second RNAseq profiling experiment aimed to identify the transcriptional signature of qRGs, aRGs and aNPs, which distinguish these properties: quiescence of qRGs; and stemness of qRGs and aRGs (Fig. S2). Progenitor cells were FACS sorted from the pallium of double transgenic *her4:drfp;mcm5:egfp* adult fish, to recover RFP-positive qRGs, RFP/GFP-double positive aRGs and GFP-positive aNPs (Fig. 3A-D). PCA analysis on biological replicates confirmed that cell type was the primary discriminative determinant of the recovered transcriptomes (Fig. 3E) and GO-term analyses of the recovered differentially expressed genes (DEGs) in the three independent comparisons further highlighted expected differentially regulated pathways: e.g. top upregulated pathways in qRGs versus aRGs include astroglial development and functions, whereas downregulated pathways include DNA replication/damage and cell cycle control genes, and genes involved in nervous system development (Fig. S4A-C, Tables S5-S10). Likewise, upregulated pathways in NSCs (qRGs or aRGs) compared with committed progenitors (aNPs) are related to glial cell development or stem cell differentiation, whereas aNPs are enriched in pathways controlling cell differentiation, together with active cell metabolism and signal transduction. Finally, we found that the pathways upregulated/downregulated in zebrafish pallial qRGs versus aRGs significantly match those recovered in two recent mouse studies between corresponding cell types [13-20% and 33-40% identical genes (Martynoga et al., 2013 and Codega et al., 2014, respectively) and 15 and 10% enriched pathways]. Values of similar order are obtained when the two mouse studies are compared with each other (17-27% of identical genes between the two studies within the sets of upregulated genes in qNSCs versus aNSCs, 24-40% for downregulated genes, and 19-35% for enriched pathways) (Tables S11 and S12, Fig. S5, and data not shown), further validating our approach and the correspondence between zebrafish and mouse adult NSC states.

The intersections of the different two-by-two comparisons of DEGs between cell types highlight several gene categories and their proposed biological interpretation (Fig. 3F, Fig. S4D-I′). Specifically, 37 genes differentially expressed between all three cell types (Fig. 3F, centre of the diagram) highlight markers that sign both the quiescent versus proliferating status and lineage progression (interpreted as transiting from stemness to commitment between RGs and NPs, see Fig. 1B). This gene set notably includes Notch and Hh signalling components, such as *notch3*, *her4*, *her8*, *hip* and *boc* (Fig. S4D). Other genes differentially expressed between qRGs and aRGs (but not between all three cell types) highlight the ‘quiescent versus proliferating’ distinction (Fig. 3F, green overlay, 96+262+221 genes). In this gene set, ‘quiescence’ hallmarks include known quiescence or stem cell marker genes, such as *bmp7b*, *pou4f2*, *lin7a* and *prom1b/2*; in contrast, the activated state is associated with genes linked with Notch signalling within populations of dividing neural progenitors, such as *ascl1a*, *neurog1* and *nestin*, genes that encode Notch ligands (*dla*, *dlb*, *dlc*, *dld*, *dll4*), *notch1b* and *her* genes (*her2*/*4.2*/*13*/*15*). The aRG state is also associated with Fgf receptors (*fgfr1*/*4*) and, as expected, cell cycle component genes (*Pcna*, *mcm4*/*5*/*6, mki67*) (Fig. S4E-G). Finally, remaining genes differentially expressed between qRGs and aNPs reflect cell stemness or commitment (Fig. 3F, pink overlay, 596+479 genes). In this gene set, we find ‘stemness’-associated genes to encode known stemness factors such as Id1 and Sox2, but also Notch effector genes (*her4.1*, *her9* and *hey1*) (Fig. S4H,I).

**Fig. 4.**
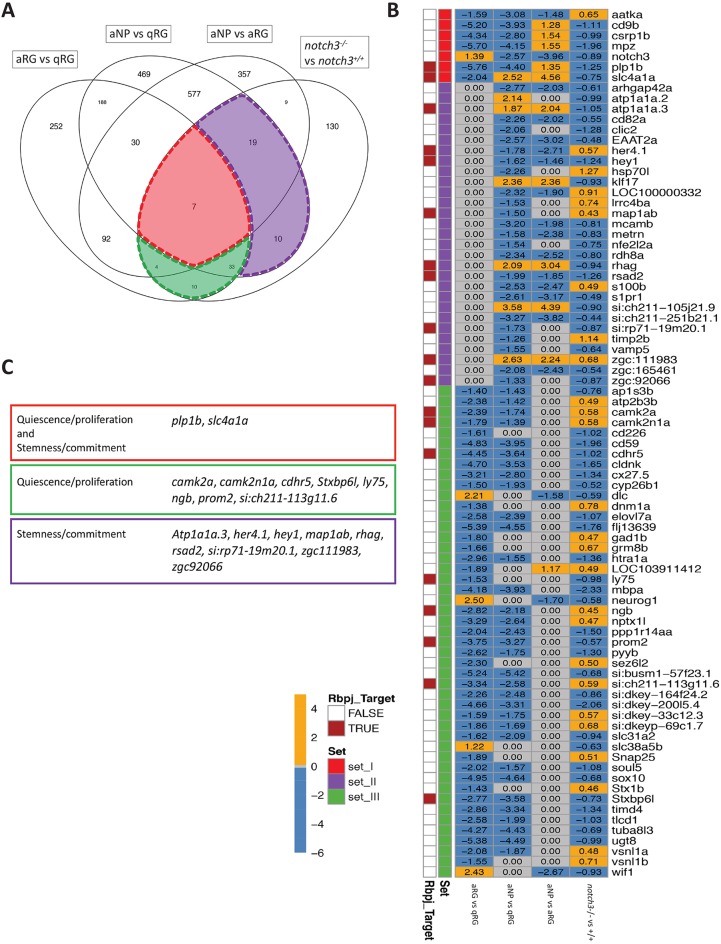
**Potentially direct Notch3 targets identify stemness- and quiescence-related genes.** (A) Venn diagram illustrating the relative distribution of differentially expressed genes recovered between cell state comparisons (Tables S5-S7) and *notch3^+/+^* and *notch3^−/−^* RGs (Tables S1 and S2). Red, transcripts related to both quiescence/proliferation and stemness/commitment; green, transcripts related to quiescence/proliferation; purple, transcripts related to stemness/commitment. (B) Heat map depicting expression of the genes identified in the three relevant categories in A (sets I-III, colour-coded as in A), with labelling of the potentially direct Notch3 targets (left column, red: with a potential RBPJ-binding site, Fig. 2E,F). (C) Potentially direct Notch3 targets belonging to the three relevant gene categories defined in A (colour-coded as in A and in B, left column).

Next, to attribute *in silico* a biological meaning to Notch3 targets in pallial RGs, we intersected Notch3-related DEGs (Fig. 2, Tables S1 and S2) with the biological gene signature of RG states (Fig. 3F, Tables S5-S7). A total of 83 differentially expressed genes (37% of all DEGs) between *notch3^+/+^* and *notch3^−/−^* RGs were found to belong to the three biological categories defined above (Fig. 4A), among which 19 were potentially direct Notch3 targets. Interestingly, although seven of the latter DEGs belong to the gene category associated with changes in both quiescence and stemness (including *notch3* itself), all others are predicted to be linked with quiescence control alone or stemness alone (Fig. 4A-C). These results suggest that the dual function of Notch3 signalling, i.e. controlling both RG quiescence and stemness, could be mediated by distinct direct cellular effectors.

### *hey1* is expressed in RGs under Notch3 control and maintains proliferating neural progenitors

To test the above hypothesis, we addressed *in vivo* the function of a predicted ‘stemness-specific’ effector of Notch3 signalling in adult RGs. We chose the *hey1* gene, as it encodes a bHLH transcription factor of the E(spl) family that was identified as a direct Notch target [in smooth muscle cells (Maier and Gessler, 2000; Iso et al., 2002) and in skeletal muscle satellite cells (Castel et al., 2013)] and acts downstream of Notch3 [in the vascular system (Zaucker et al., 2013)]. Its overexpression extends the maintenance of proliferating neural progenitors in the mouse embryonic neural tube (Sakamoto et al., 2003), and Hey1 function is necessary for proper embryonic neurogenesis in dorsal root ganglia (Mukhopadhyay et al., 2009), but its role in adult NSCs had not yet been analysed.

**Fig. 5.**
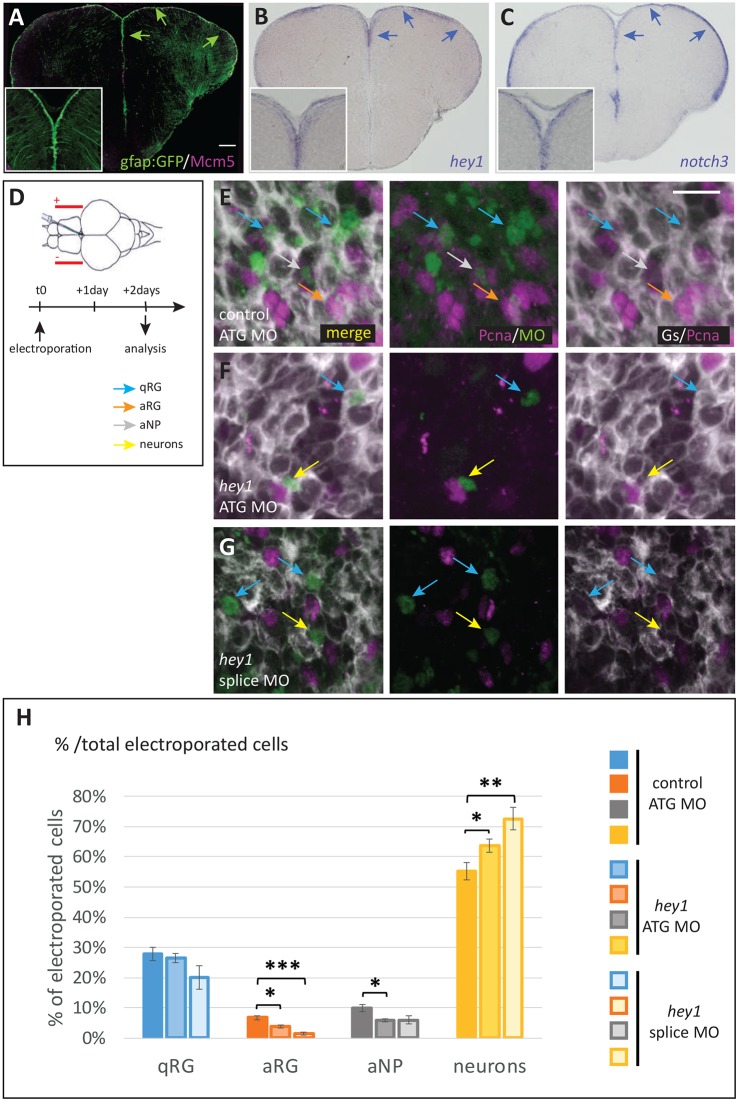
**Hey1 activity maintains the proliferating progenitor state in the adult pallial germinal zone.** (A) Cross-section of a *gfap:gfp* transgenic pallium at a mid-anteroposterior level, double immunostained for GFP (RGs) and Mcm5 (proliferating cells). Progenitor cells (qRGs, aRGs and aNPs) are confined to the ventricular zone (arrows, see high magnification inset). (B,C) Expression of *hey1* and *notch3* revealed by *in situ* hybridization on cross-sections of the adult pallium (same level as in A). Expression is confined to the VZ (arrows). (D) Experimental scheme to assess Hey1 function. *hey1* (or control) fluorescein-labelled morpholinos (MO) are injected into the brain ventricle and electroporated. The fate of MO-inheriting cells (fluorescein-positive) is assessed 2 days post-electroporation. (E-G) Representative examples of triple immunostaining to reveal cell states in cross-sections of electroporated pallia [green, fluorescein; grey, glutamine synthase (RGs); magenta, Pcna (proliferating cells)]. Examples of cell types are indicated with colour-coded arrows, as defined in H. Scale bars: 50 μm in A-C; 10 μm in E-G. (H) Quantification of cell state/type changes following Hey1 blockade. The proportion of each cell state/type within the MO-inheriting population is plotted. The proportion of neurons is significantly increased upon Hey1 blockade, whereas the proportion of proliferating cell types (aRGs and aNPs) is significantly decreased. The proportion of qRGs is unchanged [*hey1* ATG MO versus ATG control MO, *P*=0.59; *hey1* splice MO versus ATG control MO, *P*=0.09 (after Holm's correction)]. Number of cells counted per brain: 196-796 for control MO, 137-413 for *hey1* ATG MO and 49-493 for *her1* splice MO. *n*=3-5 brains per condition. *P<0.05, **P<0.01, ***P<0.001.

We first verified that *hey1* expression was confined to neural progenitors in the adult (Fig. 5A,B) and embryonic (Fig. S6A,B,F) pallium, in a profile highly reminiscent of *notch3* (Fig. 5C, Fig. S6D,E), and was downregulated in *notch3^−/−^* RGs (Fig. S6C). To assess Hey1 function in the adult pallial VZ *in vivo*, we needed a conditional method and designed two fluorescein-tagged morpholinos (MOs) targeting the ATG or the second donor splice site of *hey1* transcripts, and a control MO harbouring 5 mismatches compared with the *hey1* ATG MO. We verified their efficiency and selectivity by showing that both *hey1* MOs mimicked the effect of the *hey1* mutation on pituitary development in 72 h post-fertilization (hpf) larvae (Nakahara et al., 2016), whereas the control MO had no effect (Fig. S7A-I). To assess Hey1 function in adult pallial progenitors, MOs were microinjected into the cerebral ventricle of anesthetized adult fish and electroporated, targeting ventricular cells (Fig. 5D), and the fate of MO-inheriting cells was analysed 2 days post-electroporation (Fig. 5E-H). Overall, as observed at 72 hpf (Fig. S7I), the *hey1* splice MO was more efficient than the *hey1* ATG MO, but both MOs produced the same significant results: compared with control-electroporated cells, we found that the proportion of neurons was significantly increased upon abrogating Hey1 function, at the expense of aRGs and aNPs; the proportion of qRGs, in contrast, was unchanged (Fig. 5H). These results indicate that Hey1 is necessary for the maintenance of proliferating pallial neural progenitors (aRGs and aNPs), most likely to prevent their premature generation of differentiated neurons.

### *hey1* maintains stemness characteristics in quiescent pallial NSCs

We were surprised to observe no apparent effect of Hey1 abrogation on qRGs (Fig. 5H), which normally express *hey1* at measurable levels (Fig. S4I). In contrast, overexpressing Hey1 in adult RGs *in vivo* by electroporation of a *pCMV5:hey1-P2A-nlsgfp* construct decreased the proportion of aRGs among GFP-positive cells compared with electroporation of *nlsGFP* alone (Fig. S8). Under overexpression conditions, however, Hey1 may mimic the effect of another E(spl) factor. In support of this interpretation, an analysis of RG proliferation in 7 dpf *hey1^−/−^* mutants (using both PCNA and BrdU as markers) revealed no proliferation phenotype (Fig. S7K-N), confirming the apparent lack of effect of *hey1* knockdown in adult RGs. We therefore worked to understand this apparent lack of phenotype. Compensatory genes may be expressed in qRGs, but we found that the closest *hey* gene, *hey2* (Winkler et al., 2003), was expressed at very low levels in qRGs, undetectable by *in situ* hybridization (Figs S4H′ and S6G). Instead, we therefore considered an alternative hypothesis: that *hey1*-depleted qRGs may have lost stemness. Indeed, in the absence of a positive marker for cellular quiescence, qRGs and fully differentiated, non-progenitor RGs would not be distinguished in our experiments.

**Fig. 6.**
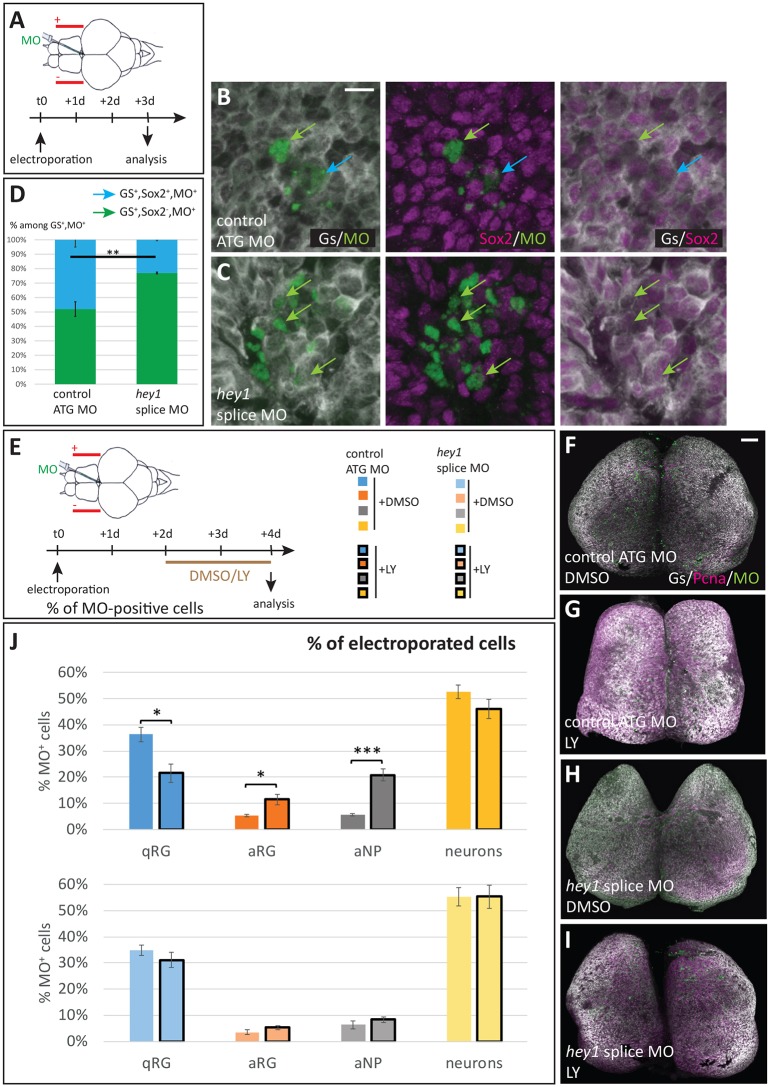
**Hey1-depleted RGs lose stemness characters.** (A-D) Effect of Hey1 abrogation on Sox2 expression in adult RGs. (A) Experimental scheme: fluorescein-tagged MOs (control MO or *hey1* splice MO) are electroporated into the pallial VZ and Sox2 expression is analysed after 3 days. (B,C) Examples of electroporated VZ double-immunostained for Gs (white) and Sox2 (magenta), with MO-containing cells in green. Colour-coded arrows indicate the different cell types (see D). (D) Quantification of the proportion of Sox2-positive (blue) and -negative (green) RGs within MO-electroporated cells. (E-J) Effect of Hey1 abrogation on RG reactivation potential. (E) Experimental scheme: fluorescein-tagged MOs are electroporated into the pallial VZ and LY411575 (or the vehicle DMSO) is applied into the swimming water between 2 and 4 days post-electroporation. RG proliferation is analysed at 4 days. (F-I) Representative examples of whole-mount electroporated/LY-treated brains double immunoprocessed for Gs (white) and PCNA (magenta). (J) Quantification of the proportions of the different cell types (colour-code indicated in E). LY treatment induces RG activation (decrease in the proportion of qRGs, increase in the proportion of aRGs) upon electroporation of the control MO, but is without effect when Hey1 function is abrogated. Number of cells counted per brain: 99-293 for control MO treated with DMSO; 153-298 for splice MO treated with DMSO; 98-262 for control MO treated with LY; and 151-353 for splice MO treated with LY. *n*=3-5 brains per condition. *P<0.05, **P<0.01, ***P<0.001, all pairwise comparisons were adjusted for multiple comparisons following the Holm's procedure. Scale bars: 10 µm in B,C; 70 µm in F-I.

To support this hypothesis, we first assessed whether *hey1* abrogation would impair expression of the stemness marker Sox2 in qRGs. Upon electroporation of the control MO, we found that around 50% of Gs-positive, MO-inheriting pallial qRGs express Sox2 (Fig. 6A,B,D). This is lower than the proportion of Sox2-positive Gfap-positive RGs under physiological conditions (around 90%, Fig. S10A), indicating a possible bias in electroporated target cells (possibly related to the size of the NSC apical surface). Upon *hey1* abrogation, this proportion dropped to 22% (Fig. 6C,D), indicating that a majority of stem cell qRGs may have lost their progenitor potential in the absence of Hey1 activity.

To further assess this possibility, we functionally assessed the reactivation capacity of NSCs upon *hey1* abrogation. We used the conditional, short-term downregulation of Notch signalling with γ-secretase inhibitors as a well-characterized reactivation paradigm (Chapouton et al., 2010; Alunni et al., 2013). Importantly, we had observed that *hey1* expression itself was not noticeably affected following a 48 h treatment with the γ-secretase inhibitor LY411575 (LY), as assessed by *in situ* hybridization (Fig. S6H,I), indicating that the treatment itself would not interfere with Hey1 function. Further, *hey1^−/−^* larvae do not display any morphological abnormalities (Nakahara et al., 2016), contrasting with larvae in which Notch signalling has been inhibited, which suffer visible defects in *hey1*-expressing organs, notably the vasculature and nervous system (e.g. Quillien et al., 2014; Itoh et al., 2003). These observations refute the fact that lowering Hey1 activity renders qRGs insensitive overall to Notch signalling and, hence, to the activation-promoting effect of LY treatment. In addition, we found that a brief LY treatment had no effect on Sox2 expression (Fig. S10A,B), suggesting no immediate effect on stemness. Together, these conditions justify our approach. Two days after MO electroporation into pallial ventricular cells, adult fish were subjected to LY (or DMSO control) treatment for a further 48 h, and the proliferation state of MO-receiving cells was analysed (Fig. 6J). The effect of *hey1* MO was no longer apparent in DMSO-treated embryos at the time of analysis (i.e. 4 days after MO electroporation, in agreement with the transient MO stability also observed in other studies, Katz et al., 2016); however, it was prominent at the onset and at least during the first day of DMSO/LY application (Figs 5H and 6D), i.e. our experimental schedule. Upon electroporation of the control MO, we found that LY treatment increased the proportion of activated progenitors, at the expense of qRGs (Fig. 6E-G,J), as expected (Chapouton et al., 2010; Alunni et al., 2013). In striking contrast, however, LY treatment had no effect on ventricular cells following *hey1* abrogation (Fig. 6E,H-J). These results indicate that, upon inactivation of Hey1 function, qRGs become insensitive to the activation-promoting effect of a transient Notch signalling abrogation. Together with the Sox2 data above, we propose that qRGs display decreased progenitor potential in the absence of Hey1 activity.

## DISCUSSION

Distinguishing the primary effect of NSC regulators, and in particular Notch signalling, on quiescence and/or stemness, has proven a difficult task as both phenotypes can occur concurrently and activation frequency itself may condition NSC lifespan. In addition, the first step in functionally assessing qRGs stemness is to test their reactivation potential, i.e. a proliferation response. In previous work, we showed increased RGs activation upon notch3MO electroporation, which is also the prominent phenotype resulting from LY treatment (presumably primarily affecting Notch3 signalling, the sole Notch receptor expressed in qRGs) (Alunni et al., 2013). Although these data readily illustrate RG quiescence control by Notch3, no clear conclusion could be drawn on stemness control. Not all qRGs respond to Notch3 blockade by reactivation (50% of RGs remain non-proliferating at 2 days post-electroporation of notch3MO, and around 10% remain non-proliferating after 5 days of LY treatment) (Alunni et al., 2013). Remaining cells may be insensitive to Notch3 levels alone at this particular time/state point, may express higher levels of Notch3 signalling that are not fully abrogated by the treatments above, or may have, instead, entered a state of irreversible cell cycle exit upon *notch3* loss of function.

The present work is important as it brings several phenotypical and molecular arguments that (1) extend the function of Notch3 in NSCs to include a direct control of NSC stemness and (ii) demonstrate that the dual activity of Notch3 on NSC quiescence and stemness occurs, in part, via independent rather than interdependent routes. Our analysis of RG fate in the pallium of *notch3^−/−^* larvae indicates that the absence of Notch3 function is initially associated with an increased division frequency, visible starting at 7 dpf, followed within 2 days by decreased proliferation and a bias towards neuronal differentiation (Fig. 1). We consider it unlikely that the initial RG proliferation burst (or delay in quiescence entry) is a sufficient event in itself to exhaust RG progenitor potential. The duration of a complete RG cell cycle at 7 dpf can be inferred to ∼18 h (Fig. 1D,F), indicating that a maximum of three cycles occurs before the change in RG fate is observed in mutants. In striking contrast, under physiological conditions, larval RGs are normally fated to long-lasting cell division, in most cases until adulthood to generate adult pallial RGs (Dirian et al., 2014). These results together argue for the quiescence loss and exaggerated differentiation of RGs in *notch3^−/−^* mutants to be distinct phenotypes. The second strong argument in favour of an independent control of NSC quiescence and stemness by Notch3 signalling is the function, identified here, of the Notch3 target Hey1. Our results indicate that *hey1* expression is confined to all progenitor cells (qRGs, aRGs and aNPs) in the adult pallium irrespective of their proliferation status, and decreases as commitment progresses within the adult pallial neurogenic lineage from qRGs to aNPs. Similar observations hold in the adult mouse hippocampal NSC lineage (F. Guillemot, personal communication). We further show that the conditional abrogation of Hey1 function in the adult pallial VZ leads to increased neuronal differentiation at the expense of activated progenitors (aRGs and aNPs), and impacts the progenitor potential of qRGs, as revealed by their loss of Sox2 expression and their incapacity to re-enter the cell cycle 48 h after LY treatment. Although the exact cell fate transitions involved in these phenotypes have not been directly traced, our proposed interpretation is the most economical in terms of lineage transitions, and reflects the rapid effects of *hey1* knockdown. One drawback of the conditional, MO-based loss-of-function strategy used in this paper is its transient efficacy. For this reason, we could not test the effect of long-term *hey1* abrogation on adult RGs, which would be necessary to fully assess stemness (including long-term self-renewal and differentiation capacity). Importantly, however, no proliferation increase was observed in the Hey1-depleted RG population, whether in *hey1^−/−^* larvae or upon conditional *hey1* abrogation at adult stage, showing that Hey1 controls the stemness/progenitor state independently of quiescence control.

The direct targets of Hey1 activity, in turn, remain to be identified. Hey1 is a transcriptional inhibitor, suggesting that its positive effect on Sox2 expression is indirect. Previous work identified the cyclin-dependent kinase inhibitor p21 as a direct negative regulator of *Sox2* transcription in the adult mouse SEZ (Marqués-Torrejón et al., 2013). Interestingly, the conditional knock-out of *p21* in mouse induces NSC growth arrest or premature NSC differentiation into astrocytes (Porlan et al., 2013). Whether Hey1 regulates p21 expression remains to be tested. Of note, we identified a putative Hey1-binding site at position −914 to −905 bp of the *p21* (*cdkn1a*) locus (data not shown). Sox2 is itself a direct Notch (RBPj) signalling target in adult mouse hippocampal NSCs, although its expression also integrates additional inputs (Ehm et al., 2010). Unlike the mouse *Sox2* gene, zebrafish *sox2* does not harbour recognizable RBPj-binding sites within 2 kb of its upstream region (not shown). Its expression is, however, selective to RGs, as opposed to further committed aNPs, in the zebrafish adult pallium (Fig. S4I). *sox2* expression in this context may result from several inputs, including the blockade of an inhibitor by Hey1, which is itself downstream of Notch3. In addition, Hey1 abrogation affects Sox2 expression in only some RGs (Fig. 6D) whereas it prevents RG activation with a higher efficiency (Fig. 6J). These observations may reflect the slightly different design of our experiments (read-out at 4 days post-electroporation in the activation test to permit LY activity), but could also indicate that Hey1 function distinguishes Sox2 expression from immediate reactivation potential. Although Sox2 expression is associated with NSC stemness in all systems studied (Graham et al., 2003; Suh et al., 2007; Codega et al., 2014; Favaro et al., 2009), the significance of Sox2-negative RGs at the adult pallial VZ remains to be analysed, and we found no specific preference for Sox2-positive versus -negative qRGs to be reactivated upon a short LY treatment (data not shown).

How individual RGs distinctly read and interpret the two different outputs of Notch3 signalling is an important issue that remains to be resolved. Our previous work has shown that RGs could be reactivated during a week by LY treatment, corresponding to several extra cycles, and nevertheless resume a normal fate and long-lasting NSC activity upon the end of treatment. These observations show that episodes of ‘low Notch’ sufficient to induce RG activation do not measurably impair stemness, and that the two Notch3 activities are not read as alternative fates but are rather overlapping. Thus, as a correlate to activating a partially distinct set of target genes with specific quiescence- or stemness-promoting functions, as shown here, we also propose that the Notch signalling thresholds of the downstream gene sets are different. For example, efficiency of the quiescence cascade may require higher levels or more sustained expression of Notch3 than the stemness cascade, allowing the modulation of the qRGs↔aRGs transition without affecting stemness. Alternatively, or in addition, different cellular sub-states that a qRG cell transitions through may differentially alter its sensitivity to changes in the quiescence or the stemness cascades. Overall, a dose-dependent response of adult NSCs to Notch3 for the key cell state choices ‘quiescence or proliferation’ and ‘progenitor or differentiation’ is strongly reminiscent of the scenario proposed to control endocrine progenitor fate in the zebrafish embryonic pancreas (Ninov et al., 2012). The uncoupling between these processes is further enhanced in our case by the fact that, unlike in the pancreas, differentiation would not be necessarily preceded by cell cycle re-entry. The dose-dependency scenario is in further agreement with the observation that *hey1* expression is initially not affected by LY treatment for 48 h (Fig. S6), and with the fact that *hey1* requires lower Notch signalling activity than other targets, e.g. *Hes/her* genes in other systems such as the inner ear (Neves et al., 2011; Petrovic et al., 2015). This lowered reliance of *hey1* expression on Notch activity could also result from its synergistic transcriptional control by additional signalling pathways (Lau et al., 2016). In this context, it is interesting to note that muscle satellite cells, which, like NSCs, are maintained into quiescence in a Notch-dependent manner, can also respond to Notch blockade in two alternative ways: cell cycle re-entry or direct differentiation (Mourikis et al., 2012). Furthermore, *Hey1* is a Notch target in muscle satellite cells – which also express Notch3 – and inhibits myogenic differentiation when overexpressed. These findings suggest that the Notch targets or contexts that differentially translate Notch signalling into quiescence or stemness control in adult NSCs may be shared with muscle satellite cells, and it will be interesting to directly test this hypothesis.

The present study offers, in addition to Hey1, a series of candidate Notch3 effectors for the control of quiescence or stemness, or both, that can be the subject of future functional assays. In addition, our data also permit the first direct molecular comparison of pallial zebrafish quiescent versus activated NSCs with their mouse counterparts. Although isolated based on different markers or experimental schemes [distinction of mouse quiescent and activated NSCs based on their response to BMP signalling in culture (Martynoga et al., 2013) or their expression of GFAP/prominin versus GFAP/prominin/EGFR (Codega et al., 2014), and distinction of qRGs and aRGs based on their expression of reporter proteins driven by the promoters of *her4*/MCM5 versus *her4* alone in the present study], the percentage of identity between recovered genes is equivalent in two-by-two comparisons of the gene sets (Tables S11 and S12), and commonly upregulated pathways in aNSC/RGs between the three studies highlight biological processes that suggest equivalent positions in the neurogenic lineage: in addition to cell cycle-related processes, aNSCs and RGs appear enriched in genes associated with neuronal commitment or differentiation, a response to EGF and FGF signalling, and Notch signalling pathway involved in fate commitment (Table S12). The number of genes commonly upregulated in qNSCs and RGs between the three studies is too low for a meaningful interpretation, but, at the pathway level, similar biological processes are enriched in the different data sets (Table S13). These results further validate the molecular similarities existing between mouse and zebrafish adult NSCs at the population level under physiological conditions, in addition to sharing common activation pathways. It is likely that these similarities will appear even more prominent when NSC heterogeneity is fully understood, and the current development of single cell ’omics methods will help in this endeavour.

## MATERIALS AND METHODS

### Zebrafish lines and genotyping

Three- to 9-month-old adults or juveniles of the wild-type AB zebrafish strain, the transgenic lines *Tg(Gfap:gfp)^mi2001^* (referred to as *Gfap:gfp*) (Bernardos and Raymond, 2006), *Tg(her4.3:dRFP)* (referred to as *her4:dRFP*) (Yeo et al., 2007) and *Tg(mcm5:eGFP)^gy2^* (referred to as *mcm5:gfp*) (Dray et al., 2015), and the *notch3^fh332^* null mutant allele (Alunni et al., 2013) were used. Seven- to 10-day-old *notch3^fh332/fh332^* mutant larvae and their *notch3^+/+^* wild-type siblings were obtained by intercrossing *notch3^fh332/+^* adult zebrafish. Genotyping of *notch3^fh332^* carrier fish was performed as previously described (Alunni et al., 2013). *hey1* mutants were obtained from Dr Y. Kikuchi (Hiroshima University, Japan). The published *hey1^ha7^* allele harbours a 7 bp deletion, causing a frameshift at amino acid 7 (Nakahara et al., 2016). Upon sequencing, we found a new different allele, harbouring an 11 bp deletion, leading to a frameshift and the production of a truncated protein (Fig. S9). This allele was used in this study.

### *In situ* hybridization

Whole adult brains or embryos were incubated at 65°C for 18 h in 2 ng/μl DIG-labelled mRNA probes for *notch3* (Itoh et al., 2003), *her4.1* (Takke et al., 1999), *hey1* and *hey2* (see below), or *pomca* and *gh* (Nakahara et al., 2016), then with POD-conjugated anti-DIG (sheep, Roche, 1:500). Dissected juvenile brains were hybridized with a DIG-labelled probe, incubated with anti-DIG-AP Fab fragments (Roche) and then cryosectioned. All *in situ* hybridization experiments used NBT/BCIP (Sigma). Partial cDNA sequences for *hey1* and *hey2* were amplified from adult brain cDNA using the Takara La Taq Polymerase (Takara) with the following primers: *hey1* forward, 5′-GCAGAGACTGCACGTTACCTC-3′; *hey1* reverse, 5′-GCCCCTATTTCCATGCTCCAG-3′; *hey2* forward, 5′-GACTGAAGTGGCCAGGTATTTG-3′; *hey2* reverse, 5′-GCTCCCGCTGCTCTGTTGGGATG-3′. The PCR fragments were subcloned using the StrataCone PCR Cloning kit (Stratagene).

### Immunohistochemistry

Whole brains were fixed overnight in 4% paraformaldehyde in PBS and kept in 100% methanol at −20°C. Following rehydration, brains were either embedded in 3% agarose blocks and vibratome sectioned (50 µm) or processed for whole-mount immunohistochemistry. An antigen-retrieval step was performed for BrdU and/or Pcna immunolabelling: for BrdU, sections were incubated in 2 M HCl at room temperature for 30 min; for Pcna, brains were incubated in Histo-VT One (Nacalai Tesque) for 60 min at 65°C. The following primary antibodies were used: MCM5 (1:500, kindly provided by Soojin Ryu, Max Planck Institute for Medical Research, Heidelberg, Germany), anti-BLBP (1:1000, rabbit, Millipore, ABN14), anti-PCNA (1:250, mouse IgG2a, Santa Cruz Biotechnology, sc56; 1:500, rabbit, Genetex, GTX124496), anti-BrdU (1:150, rat IgG1, Abcam, ab6326), anti-GFP (1:1000, chicken, Aves laboratories, GFP 1020), anti-glutamine synthetase (1:1000, mouse IgG2a, Millipore, MAB302), anti-dsRed (1:250, rabbit, Clontech, 632496), anti-Sox2 (1:500, rabbit, Abcam, ab97959; 1:200, mouse IgG1, Abcam, ab171380) and anti-active Caspase-3 (1:300, rabbit, BD Pharmigen, 559565). Secondary antibodies raised in goat coupled to AlexaFluor dyes (Invitrogen) were used (1:1000).

### BrdU pulse-chase

Seven days post-fertilization juveniles were distributed into a six-well plate at a density of around 10 per well in 10 mM BrdU (Sigma) solution in embryo medium containing 15% DMSO for 20 min on ice. Then, the embryos were transferred in EM containing 10 mM BrdU at 28°C for 5 h. 1 mM BrdU was applied to the adult fish water at 28°C in the dark. Fish were subsequently transferred to a tank with fresh water during the chase period.

### LY411575 treatments

Notch signalling was blocked using 10 μM LY411575 (LY; Stemgent) applied in the swimming water at 28°C. The LY solution was exchanged daily. Control fish were treated with the same final concentration (0.04%) of DMSO carrier.

### Ventricular micro-injections and electroporation of *hey1* morpholinos

To selectively block Hey1 function, we electroporated fluorescein-tagged ATG or splice *hey1* morpholinos (MOs) (GeneTools) or a 5-mismatch ATG control MO, into neural progenitors of the adult pallium: MOs at 1.3 mM were injected into the brain ventricle of anesthetized adults as described previously (Rothenaigner et al., 2011). The MOs used were as follows: *hey1* ATG MO, 5′-TCATTTTTCGACAGTTTAGCAGCGC-3′; *hey1* splice MO 5′-AAAAAAATGTCTTACCCCTCTGCGA-3′; *hey1* ATG control MO, TGATTTTTGGACACTTTAGCACCCC. For validation in embryos, the different MOs were injected at 0.2 mM at the one-cell stage and pituitary markers (*pomca*, *GH*) were analysed by *in situ* hybridization at 72 h post-fertilization (hpf) and compared with the phenotype of *hey1* mutants (Nakahara et al., 2016).

### *hey1* overexpression in adult RGs

The full-length coding sequence of zebrafish *hey1* was cloned from adult brain cDNA (forward primer, 5′-ATGAAGAGAAATCACGATTTCAGCTC-3′; reverse primer: 5′-GAAGGCCCCTATTTCCATGC-3′) using the Strataclone PCR Cloning kit (Stratagene). *nlsGFP* was cloned by PCR from *pME-nlsGFP-P2A* (Fowler et al., 2016) (forward primer, 5′-ATGGCTCCAAAGAAGAAGCG-3′; reverse primer, 5′-TTACTTGTACAGCTCGTCCATGC-3′). A Gibson Assembly (NEBuilder HiFi kit - NEB) was performed to assemble *hey1*, *P2A* and *nlsGFP* sequences in *pCMV5* linearized with *Bgl*II. As a control, the *nlsGFP* sequence alone was also inserted in *pCMV5* using *Bgl*II and *Hin*dIII. Electroporation was carried out as above after injecting *pCMV5:hey1-P2A-nlsGFP* or *pCMV5:nlsGFP* at 900 nM into the brain ventricle.

### Image acquisition and cell counting

For *in situ* hybridization, all images were taken on an Olympus VS120 stereomicroscope using a 20× air objective. For immunohistochemistry, images were taken on Zeiss LSM700 or LSM710 confocal microscopes using the following objectives: 20× air, 40× oil or 63× oil. Images were processed using Imaris 7 (Bitplane). Sections are presented as single confocal planes except for Fig. 2A,B, which correspond to maximum intensity projections of two adjacent confocal planes. Whole-mount telencephali processed for immunohistochemistry (Fig. 5E, Fig. 6B,C,F-I, Fig. S8B,C) are presented as 3D reconstructions of acquired *z*-stacks. For cell counting in Fig. 1 and Fig. S1, vibratome sections were prepared and Blbp-, BrdU- and Pcna-positive cells were counted manually on optical sections from the Dm region of the pallial VZ. For cell counting in Figs 5 and 6, whole-mount brains were prepared from a minimum of three telencephali. Gs-, Pcna- Sox2- and fluorescein-positive cells were counted manually from the entire Dm VZ. For cell counting in Fig. S7, *pomca* and *gh*-positive cells were counted manually on flat-mounted whole larval brains.

### Statistical analyses

Cell quantifications were performed on 3D image reconstructions of the dorso-medial pallium (Dm) for all *hey1* knockdown experiments and on telencephalic sections for RG phenotyping in *notch3* mutant larvae. Between one and six 1 µm optical sections (average 2.84, mode 3) corresponding to different rostrocaudal levels were analysed. Only the Dm region was investigated. For each optical section, cell counts were normalized by Dm ventricular zone length to account for span variations along the rostrocaudal axis. They are reported as number of cells per 100 µm of ventricular zone length. Images were analysed using Imaris software (Bitplane) and investigators were not blinded to treatments/genotypes. Balanced ratios of females and males were included in the different experimental groups. Data are presented as experimental mean±s.e.m. from 3 to 11 animals per condition. Statistical analyses were carried out using InVivoStat or Microsoft Excel (Clark et al., 2012). The normality of the residuals of the responses was assessed using normality probability plots and the homogeneity of the variance was inspected on a predicted versus residual plot (Bate and Clark, 2014). When the data deviated noticeably from either criterion, they were square root transformed. In addition, all proportion responses were transformed using the arcsine function (Bate and Clark, 2014). Data displaying an approximately Gaussian distribution of residuals and homoscedastic responses were analysed using parametric tests. The statistical significance at the 5% level (α=0.05) was determined either with a two-tailed independent (unpaired) *t*-test for single comparisons or with least significant difference test when several pairwise comparisons were made. In the latter case, *P*-values were adjusted for multiple comparisons according to the Holm's procedure, except for the RT-qPCR validation of Notch3 targets, where the Benjamini-Hochberg's procedure was used. When factors (MOs, time of chase, ages, genotypes, drug treatments) were analysed at more than two levels (control, *hey1* ATG and *hey1* splice MOs; 1-3 days of chase; *notch3^+/+^* and *notch3^−/−^*; DMSO and LY511575) or in combination, overall effects were determined by analysis of variance (ANOVA). No gateway ANOVA approach was used and pairwise comparisons were carried out independently of the results of the ANOVA. Batches, experiments, experimenters (studies of *notch3* and *hey1* mutant larvae, *hey1* knockdown experiment) and biological replicates (qPCR) were used as blocking factors. Single comparisons between responses harbouring significant deviations of their residuals from the normal distribution and/or heterogeneous variances were analysed using the non-parametric Mann–Whitney test. When experiments had to be subdivided, fish numbers were balanced between experimental groups. No computational randomization methods were used, but special attention was paid to maximize the random distribution of fish across treatments.

### Cell dissociation, FACS sorting, sample preparation and RNA sequencing

Adult brains and larval heads were dissected in Ringer's solution. Cell dissociation was carried out according to Manoli and Driever (2012) (see supplementary Materials and Methods). Cells were sorted on a FACSAria III SORP (Becton-Dickinson) cytometer. RNA was isolated with the Arcturus PicoPure Isolation kit, according to the manufacturer's protocol (Life Technologies). Three independent experiments with 20 pooled adult telencephali were performed for library construction from *her4:drfp;mcm5:gfp* double transgenic fish. At least 2.5 ng of total RNA was extracted for each biological replicate. Three independent experiments with 15 larval heads for each condition were performed for library construction from genotyped *notch3*^*fh332/fh332*^ mutant versus *notch3*^+/+^ larvae, crossed into the *Gfap:gfp* background. At least 4 ng of total RNA was extracted for each biological replicate. Libraries were constructed using the Totalscript RNAseq kit from Epicentre (discontinued), using the oligodT option, according to the manufacturer recommendations. Paired-end sequencing was performed on a NextSeq sequencer from Illumina. We used 50 bp reads in the experiments on the adult brains and 100 bp reads in the experiment on *notch3* mutant larvae. The read quality was assessed with FastQC and the mapping carried out with TopHat2. Read counting was performed with HTSeq and differential analysis with DESeq2. RNAseq data have been deposited in GEO under accession number GSE111765.

### Analysis of RNAseq datasets

#### Mapping of sequencing reads

Quality of the reads was assessed with FastQC. We aligned raw RNAseq reads to zebrafish genome assembly (Zv9) using TopHat2 [PMID: 23618408] by providing zebrafish gene model from Ensembl (V78) as the reference gene model. The number of mapped reads varied between ∼50 and 63 million pair-ends across each sample that accounted for about 75% of pair-ends reads. Because of the bias they introduce, multiple mapping reads were excluded. Unique mapping reads were selected using samtools (version 1.3.1).

#### Differential expression analyses

DESeq2 (v.1.14.1) (Love et al., 2014) Wald test was used to assess differential expression between groups. The input data are pre-filtered matrices in which no reads or nearly no reads have been removed. Each pre-filtered matrix contains the raw count data where each row indicates the transcript, each column indicates the sample and each cell indicates the number of reads mapped to the transcript in the sample. *P*-values for genes surviving independent DESeq2 filtering (see Love et al., 2014) were adjusted for multiple comparison correction using the Benjamini–Hochberg correction for FDR at a threshold of *P*<0.05 (Benjamini and Hochberg, 1995). No minimum LFC threshold was applied.

#### Identification of RBPj-binding sites on Notch3 target genes

We used the matrix-scan tool of the RSAT suite (Regulatory Sequence Analysis Tool) (Turatsinze et al., 2008) with the position weight matrix M00234 from the TRANSFACS database that was constructed based on published Su(H)-bound sequences.

#### Gene ontology enrichment analyses

Tables for each comparison from DESeq2 results were ordered by the Wald statistic values. Zebrafish Gene Symbol and Entrez Ids were added using the org.Dr.eg.db (v_3.4.0) and AnnotationDbi (v_1.36.2) Bioconductor Packages, using the Ensembltrans as keys. Human orthologs were added to the tables using the human and Zebrafish orthology tables from Zfin website (zfin.org/downloads/human_orthos.txt, downloaded June 2017). Duplicated Human Symbols were then collapsed by keeping the one with highest logFC. gmt files containing the GO gene collections (c5.mf.v6.0.symbols.gmt, c5.bp.v6.0.symbols.gmt) were downloaded from Molecular Signatures Database (software.broadinstitute.org/Gsea/msigdb, downloaded June 2017). The gene collections were used to perform enrichment analysis using two complementary approaches: First, an over-representation analysis (ORA) (Khatri et al., 2012) on differentially expressed genes was performed using one-sided Fisher's exact tests implemented in R script (R Development Core Team, 2013) with a Benjamini and Hochberg's multiple testing correction of the *P*-value. Then a gene set enrichment analysis (GSEA) [functional scoring method (FSC) (Khatri et al., 2012)] type of analysis using the runGSA function in piano R package (Väremo et al., 2013) was performed on the ranked list (see above) of genes.

#### Visual representation

Heatmaps were made using the R package pheatmap (v_1.0.8) and other visual representations (barplots) were made using the R package ggplot2 (v_2.2.1) (Wickham, 2009).

#### R session info

All analyses were performed using R (R Development Core Team, 2013) version 3.3.2 (2016-10-31), running under: OS×El Capitan 10.11.6 on the ×86_64-apple-darwin13.4.0 (64-bit) platform.

### RT-qPCR

RT-qPCR was performed on cDNA from the head (without eyes) of 7 dpf genotyped larvae, and expression of the *prkag1* gene was used for normalization. Data are reported as mean fold-change (2^−ΔΔCt^)±s.e.m. in *notch3^−+−^* relative to *notch3^+/+^* larvae. All details are provided in supplementary Materials and Methods and in Table S13.

## Supplementary Material

Supplementary information
