# Supplementary material for: Neural stem cell quiescence and stemness are molecularly distinct outputs of the Notch3 signalling cascade in the vertebrate adult brain
Source: Development. 2018 May 15;145(10):dev161034. doi: 10.1242/dev.161034 (PMC6001379; doi:10.1242/dev.161034)
Supplement: Supplementary information [file develop-145-161034-s1.pdf]

Than-trong et al.

Supplementary files

Supplementary methods

Cell dissociation and FACs sorting

Cell dissociation was carried out according to a previously published protocol [1], with the following modifications. Dissected telencephali or enucleated larval heads were recovered and pooled in FACSmax on ice, then placed on a 40 µm nylon cell strainer (BD FalconTM) previously moistened with FACSmax and squashed with the plunger of a 1 ml syringe. The dissociated cell solution was recovered into a 35 X 10 mm tissue culture dish (Falcon) on ice, transferred to a 2 ml tube and mixed by gentle pipetting. Dissociated tissues from adult brains were centrifuged at 1000g whereas those from larval head were centrifuged at 300g. After discarding the supernatant, cells were recovered in varying volumes (300-600 µl) of FACSmax containing 1 µg/ml DAPI (4',6-diamidino-2-phenylindole) and maintained on ice. DAPI was used to label dying cells and remove them upon sorting. Cells were sorted directly into 100 µl of extraction buffer with a FACSAria III SORP (Becton-Dickinson) flow cytometer equipped with a 90 µm-diameter nozzle.

RT-qPCR

Zebrafish larvae were genotyped for *notch3* alleles at 6 dpf. Genotyped larvae were then dissected at 7 dpf and 9 to 15 heads (without eyes) from either $notch3^{+/+}$ and $notch3^{fh332/332}$ larvae were pooled in phosphate buffered saline (PBS) on ice and immediately homogenised with Trizol (life technologies, 15596-026) by repeated suctions with a 1 ml syringe and a G20 needle. Homogenised samples were stored at -80°C for less than two months. Total RNA was isolated according to the manufacturer's protocol, recovered in 50 µl RNase-free water, treated with DNaseI (RNase-free DNase set, Qiagen no.79254), purified and concentrated in 14 µl RNase-free water with RNeasy minelute cleanup kit (Qiagen, no. 74204). RNA was quantified with a Nanodrop One (Thermo scientific) spectrophotometer. Three independent experiments were carried out, each with matched $notch3^{+/+}$ and $notch3^{fh332/332}$ siblings. Between 56.5 ng/µl and 148 ng/µl of total RNA, with A_{260}/A_{280} comprised between 1.95 and 2.04, were recovered from the different samples. RNA integrity was not assessed due to limited material availability. First strand cDNA was synthesized from the entire total RNA for each sample (between 694.9 ng and 1761.2 ng) with 200 units of Superscript II reverse transcriptase (Invitrogen, Cat. No. 18064-014) and 2.5 µM of random hexamer primers (Invitrogen, N8080127). Volumes were adjusted to fit a final volume of 25 µl for each reaction. cDNA was stored at -20°C.

RT-qPCR primers were designed with Primer-BLAST (NCBI website) following the recommended parameters for use with SYBR green system [2]: 18-25 bp in length, GC content of 45% - 55%, melting temperature (Tm) between 59°C and 62°C, amplicon of 80-150 bp. Primer pairs were selected such that a least one of the primers spanned an intron-exon junction. To determine the amplification efficiencies of each primer pair, we performed 5-fold serial dilutions of pre-amplified cDNA obtained in a previous test experiment (not shown), from RG total RNA. Amplification efficiencies were determined by quantitative PCR (qPCR) from the slope of curves representing the quantification cycles Cq as a function of the logarithm (Log_{10}) of cDNA input according to $Efficiency=10^{-1/slope}$ [3]. cDNA dilutions covered a dynamic range of 625 (from 1.5625 ng/µl to 0.0025 ng/µl) and quantification were done on 3 independent biological replicates, each with 3 technical replicates. An amplification efficiency of 2 +/- 10% was considered satisfactory [4]. Retained primer pairs, their amplification efficiencies, the correlation coefficient (R^2) of their calibration curves, the length of the amplicons together with their approximate locations and straddled exon-exon junctions are reported in Table S13 [5].

prkag1 was chosen as a reference gene for qPCR normalization based on both its low coefficient of variation (6%) and its measurable average expression level (34.3 transcripts per million –TPM) across the *notch3*$^{+/+}$ and *notch3*$^{fh332/332}$ samples of the RNA-seq dataset.

qPCR was performed with Power SYBR® Green PCR Master Mix (ThermoFisher Scientific, 4367659) on a QuantStudio™ 3 (ThermoFisher) thermocycler. Between 1.3 and 1.6 µl of cDNA was used per 20 µl reaction. The final concentration of each primer was 0.3 µM. The following parameters were used:

	Polymerase activation	Denaturation	Annealing / extension
**temperature**	95°C	95°C	60°C
**time**	10 min	15 sec	1 min
**Cycles**	1	40	

Melt curves were systematically generated by the end of the last PCR cycle to confirm the absence of unspecific amplification products or primer dimers. The size of the amplicons was verified by electrophoresis on a 3% agarose gel.

Expression changes of target genes between *notch3*$^{+/+}$ and *notch3*$^{fh332/332}$ larvae were analyzed with the $2^{-\Delta\Delta Ct}$ method [6][4]. Cq values for each target were normalized to *prkag1*. Each sample/target pair was run in technical triplicates and three independent biological replicates were used. For each biological replicate, the normalized expression of target genes in *notch3*$^{fh332/332}$ mutants (ΔCq) was compared with expression in their *notch3*$^{+/+}$ siblings. Data are reported as averaged fold change ($2^{-\Delta\Delta Ct}$) +/- s.e.m. relative to *notch3*$^{+/+}$ larvae. Statistical inference was carried out on normalized Cq conditions. Reported p-values were corrected for multiple comparison testing with the Benjamini–Hochberg procedure.

Development • Supplementary information

Table S1. Genes down-regulated in 7dpf *notch3*$^{-/-}$ *gfap:gfp* RG compared to *notch3*$^{+/+}$ RG.

Click here to Download Table S1

Table S2. Genes up-regulated in 7dpf *notch3*$^{-/-}$ *gfap:gfp* RG compared to *notch3*$^{+/+}$ RG.

Click here to Download Table S2

Table S3. GO terms and corresponding genes list associated with differentially expressed genes between 7dpf *notch3*$^{-/-}$ and *notch3*$^{+/+}$ RG.

Click here to Download Table S3

Table S4. Differentially expressed genes in 7dpf *notch3*$^{-/-}$ compared to *notch3*$^{+/+}$ RG harboring a putative binding site for RBPJ within 2kb from their TSS.

Click here to Download Table S4

Table S5. Differentially expressed genes between adult qRG and aRG.

Click here to Download Table S5

Table S6. Differentially expressed genes between adult qRG and aNP.

Click here to Download Table S6

Table S7. Differentially expressed genes between adult aRG and aNP.

Click here to Download Table S7

Table S8. GO terms (Biological Process) and corresponding genes list associated with differentially expressed genes between qRG and aRG (from Table S5). The list was computationally established using ontologyIndex R package and an *ad hoc* developed R script identifying hierarchically related GO terms (Columns E-G). GO terms enrichment values were tested by the Fisher (H) or Piano (I) methods. Terms plotted in Fig.S3A are highlighted in yellow. They correspond to those having the highest hierarchical value (0 ancestors), further manually curated to remove terms referring to non-nervous system-related organs, reflecting the pleiotropic activity of some molecular pathways.

Click here to Download Table S8

Table S9. GO terms (Biological Process) and corresponding genes list associated with differentially expressed genes between qRG and aNP (from Table S6). The list was computationally established using ontologyIndex R package and an *ad hoc* developed R script identifying hierarchically related GO terms (Columns E-G). GO terms enrichment values were tested by the Fisher (H) or Piano (I) methods. Terms plotted in Fig.S3A are highlighted in yellow. They correspond to those having the highest hierarchical value (0 ancestors), further manually curated to remove terms referring to non-nervous system-related organs, reflecting the pleiotropic activity of some molecular pathways.

Click here to Download Table S9

Table S10. GO terms and corresponding genes list associated with differentially expressed genes between aRG and aNP (from Table S7). The list was computationally established using ontologyIndex R package and an *ad hoc* developed R script identifying hierarchically related GO terms (Columns E-G). GO terms enrichment values were tested by the Fisher (H) or Piano (I) methods. Terms plotted in Fig.S3A are highlighted in yellow. They correspond to those having the highest hierarchical value (0 ancestors), further manually curated to remove terms referring to non-nervous system-related organs, reflecting the pleiotropic activity of some molecular pathways.

Click here to Download Table S10

Table S11. Comparison of the GO terms (Biological process) enriched in quiescent versus activated adult mouse NSCs and the present study. **Page 1. Columns A-C**: Enriched GO terms from one of the studies in columns D-G. **Columns D,E**: GO terms enrichment values in quiescent NSCs compared to activated NSCs (Table S5), tested by the ORA (D) or GSEA (E) methods. Significant enrichment is attributed value “1”, non-enrichment value “0”. **Columns F,G**: GO terms enrichment values in quiescent NSCs compared to activated NSCs from the following sources, respectively: “Martynoga” is from [7], “Codega” is from [8], tested by the ORA method. **Page 2.** Same data ordered by master biological process. Color code: enriched in all statistical analyses; yellow: enriched in at least one mouse study and one statistical method in the present work (Table S5); brown: enriched in the present work only but not in mouse NSC studies.

Click here to Download Table S11

Development • Supplementary information

Table S12. Comparison of the GO terms (Biological process) enriched in activated versus quiescent adult mouse NSCs and the present study. **Page 1. Columns A-C**: Enriched GO terms from one of the studies in columns D-G. **Columns D,E**: GO terms enrichment values in activated NSCs compared to quiescent NSCs (Table S5), tested by the ORA (D) or GSEA (E) methods. Significant enrichment is attributed value "1", non-enrichment value "0". **Columns F,G**: GO terms enrichment values in activated NSCs compared to quiescent NSCs from the following sources, respectively: "Martynoga" is from [7], "Codega" is from [8], tested by the ORA method. **Page 2.** Same data ordered by master biological process. Color code: enriched in all statistical analyses; yellow: enriched in at least one mouse study and one statistical method in the present work (Table S5); brown: enriched in the present work only but not in mouse NSC studies.

Click here to Download Table S12

Table S13. Primers used for the RT-qPCR analysis.

Click here to Download Table S13

Development • Supplementary information

References for supplementary material

1. Manoli, M., and Driever, W. (2012). Fluorescence-activated cell sorting (FACS) of fluorescently tagged cells from zebrafish larvae for RNA isolation. Cold Spring Harb. Protoc. *7*, 879–886.
2. Lan, C.C., Tang, R., Leong, I.U.S., and Love, D.R. (2009). Quantitative real-time RT-PCR (qRT-PCR) of zebrafish transcripts: Optimization of RNA extraction, quality control considerations, and data analysis. Cold Spring Harb. Protoc. *4*.
3. Pfaffl, M.W. (2001). A new mathematical model for relative quantification in real-time RT-PCR. Nucleic Acids Res. *29*, 45e–45. Available at: https://academic.oup.com/nar/article-lookup/doi/10.1093/nar/29.9.e45.
4. Schmittgen, T.D., and Livak, K.J. (2008). Analyzing real-time PCR data by the comparative C T method. Nature *3*, 1101–1108.
5. Bustin, S., Benes, V., Garson, J., Hellemans, J., Huggett, J., Kubista, M., Mueller, R., Nolan, T., Pfaffl, M., Shipley, G., *et al.* (2009). The MIQE guidelines: minimum information for publication of quantitative real-time PCR experiments. Clin. Chem. *55*, 611–622.
6. Livak, K.J., and Schmittgen, T.D. (2001). Analysis of relative gene expression data using real-time quantitative PCR and the 2(-Delta Delta C(T)) Method. Methods *25*, 402–8. Available at: http://www.ncbi.nlm.nih.gov/pubmed/11846609.
7. Martynoga, B., Mateo, J.L., Zhou, B., Andersen, J., Achimastou, A., Urbán, N., van den Berg, D., Georgopoulou, D., Hadjur, S., Wittbrodt, J., *et al.* (2013). Epigenomic enhancer annotation reveals a key role for NFIX in neural stem cell quiescence. Genes Dev. *27*, 1769–1786.
8. Codega, P., Silva-Vargas, V., Paul, A., Maldonado-Soto, A.R.R., DeLeo, A.M.M., Pastrana, E., and Doetsch, F. (2014). Prospective Identification and Purification of Quiescent Adult Neural Stem Cells from Their In Vivo Niche. Neuron *82*, 545–559. Available at: http://linkinghub.elsevier.com/retrieve/pii/S0896627314001706%5Cnhttp://www.ncbi.nlm.nih.gov/pubmed/24811379.

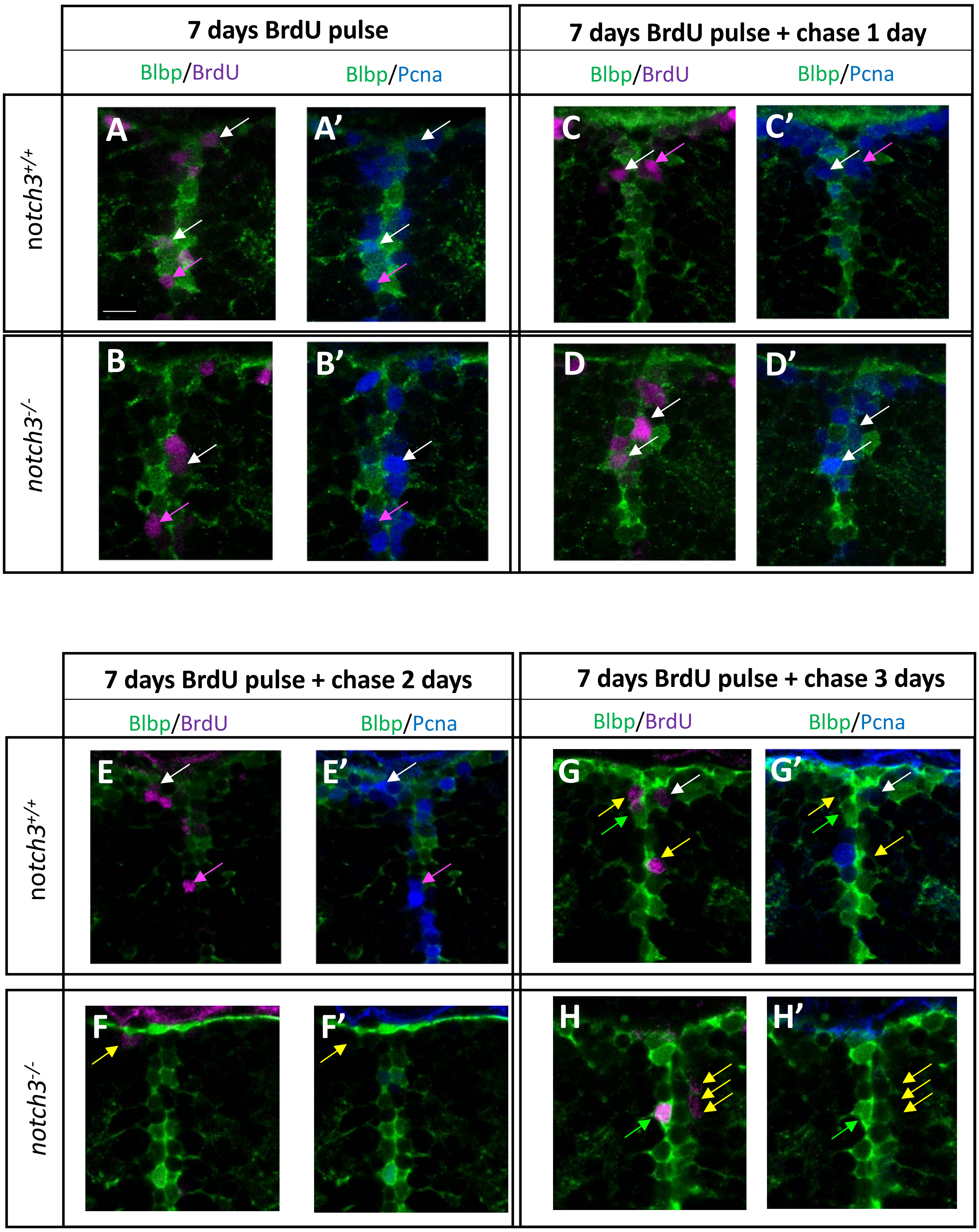

Than-Trong et al, Figure S1A-H'

Development • Supplementary information

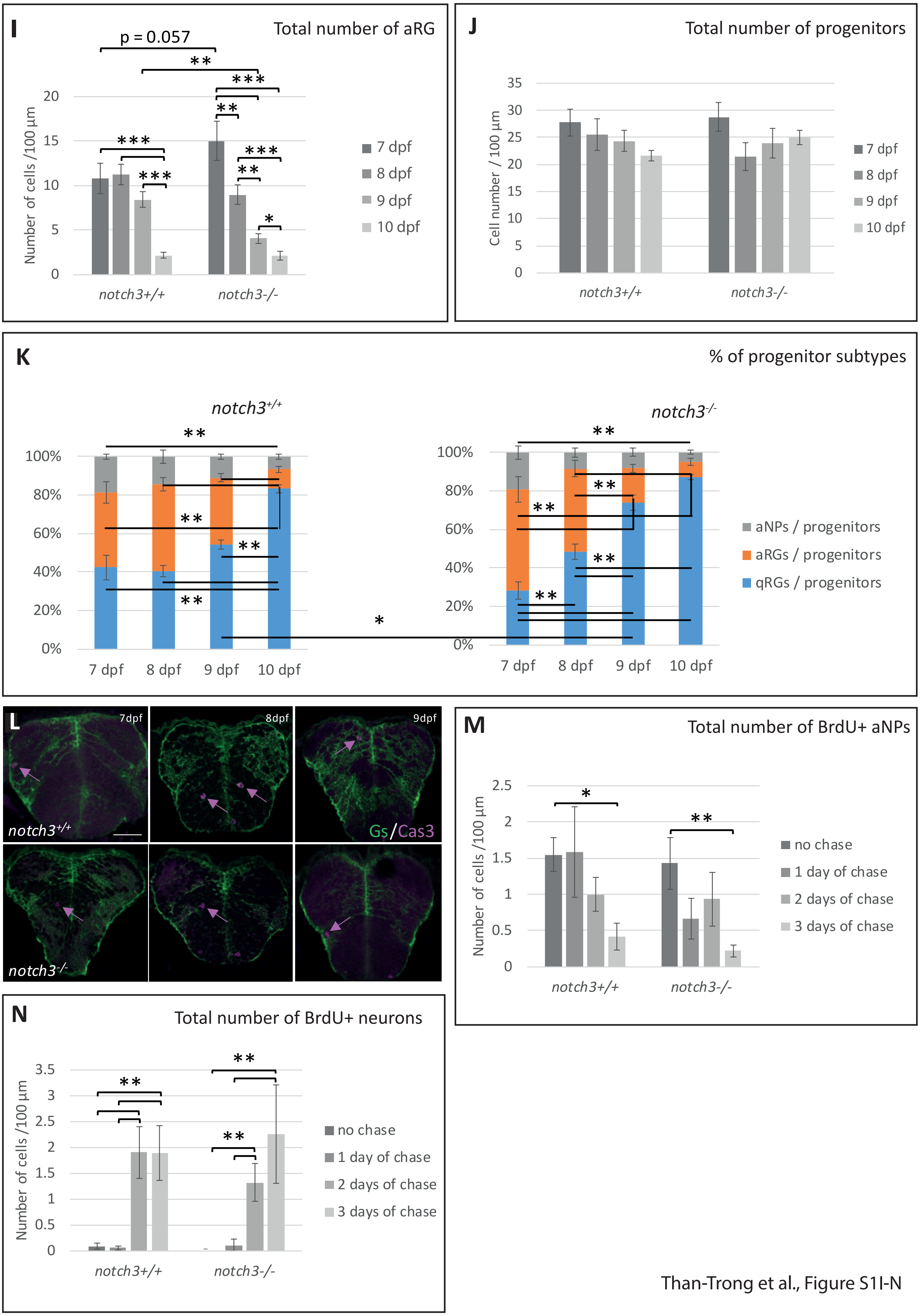

Than-Trong et al., Figure S1I-N

Development • Supplementary information

<u>**Figure S1**</u> (related to Fig.1). **BrdU tracing of neural progenitors of the 7dpf pallial VZ in *notch3*$^{+/+}$ and *notch3*$^{-/-}$ larvae. A-H'.** A BrdU pulse was applied at 7dpf (time point 0, A-B') and the identity of BrdU-positive cells was analyzed after 1 (C-D'), 2 (E-F') or 3 (G-H') days of chase by triple immunocytochemistry for BrdU (magenta), BLBP (green) and PCNA (blue). Cross-sections of the pallial VZ (same area as Fig.1A',C'). Arrows point to the different BrdU-positive cell types (green arrows: qRG, white arrows: aRG, magenta arrows: aNP, yellow arrows: neurons). Scale bar: 10μm. **I-K,M,N.** Further quantifications related to Fig.1. **I-K.** Values related to Fig.1A-E. **M,N.** Absolute values related to Fig.1G-H. **p<0.01, *p<0.05. **L.** Imunostaining for phospho-Caspase 3 (magenta) and the RG marker Gs (geen) on cross sections of the telencephalon wildtype and *notch3*$^{-/-}$ siblings at 7, 8 and 9dpf. Few Cas3-positive cells are visible (arrows), none of them within the RG population (number of counted RG per telencephalon: 70, number of Cas3-positive cells: 0, n=23 wildtype and 15 mutants). Scale bars: 40μm.

Development • Supplementary information

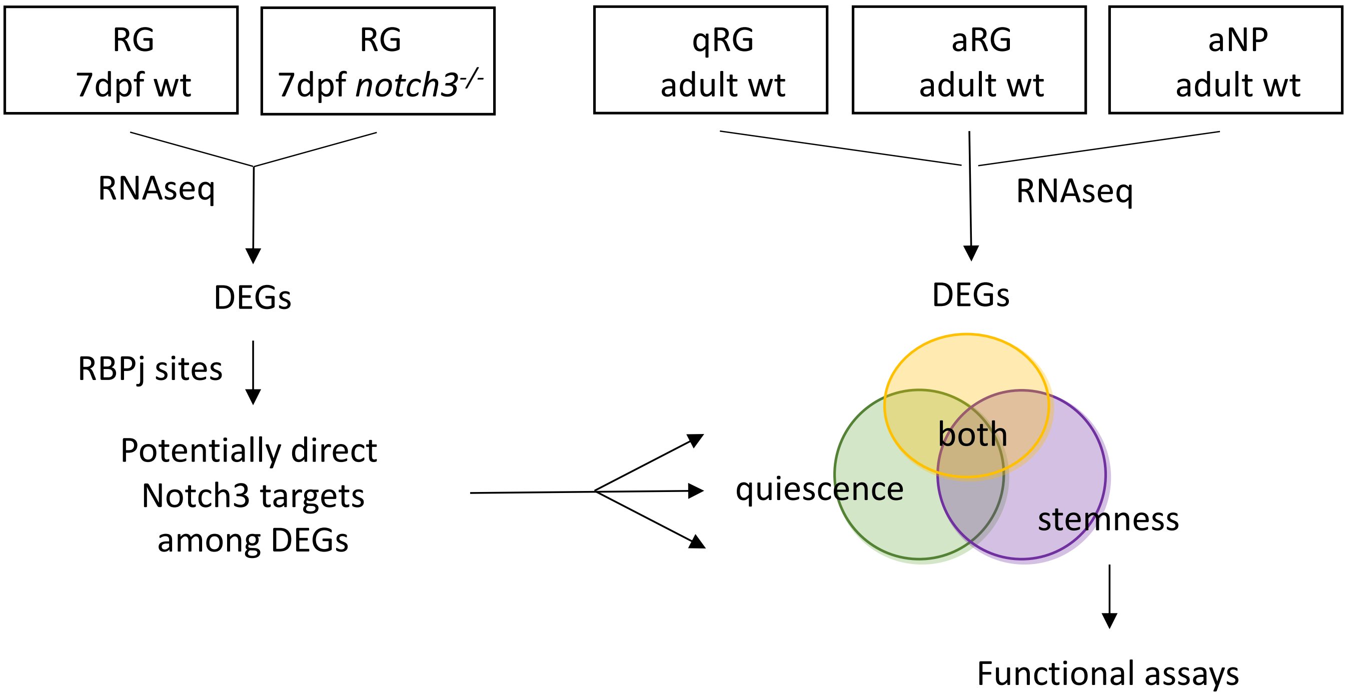

Than-Trong et al., Fig.S2

Figure S2 (related to Figs.2,3). Experimental strategy to uncover the molecular effectors of Notch3 activities in post-embryonic RG. Notch3 targets, identified from the compared RNAseq of wildtype and *notch3*$^{-/-}$ RG followed by an *in silico* search for RBPj binding sites (Fig.2), were positioned within sorted sets of genes differentially expressed (DEGs) between adult qRG, aRG and aNP (Figs.3,4). The latter comparison permits to postulate the function of the gene sets (controlling RG quiescence, stemness or both). The approach is then validated by the functional assessment of one candidate "stemness" Notch3 target (Figs.5,6).

Development • Supplementary information

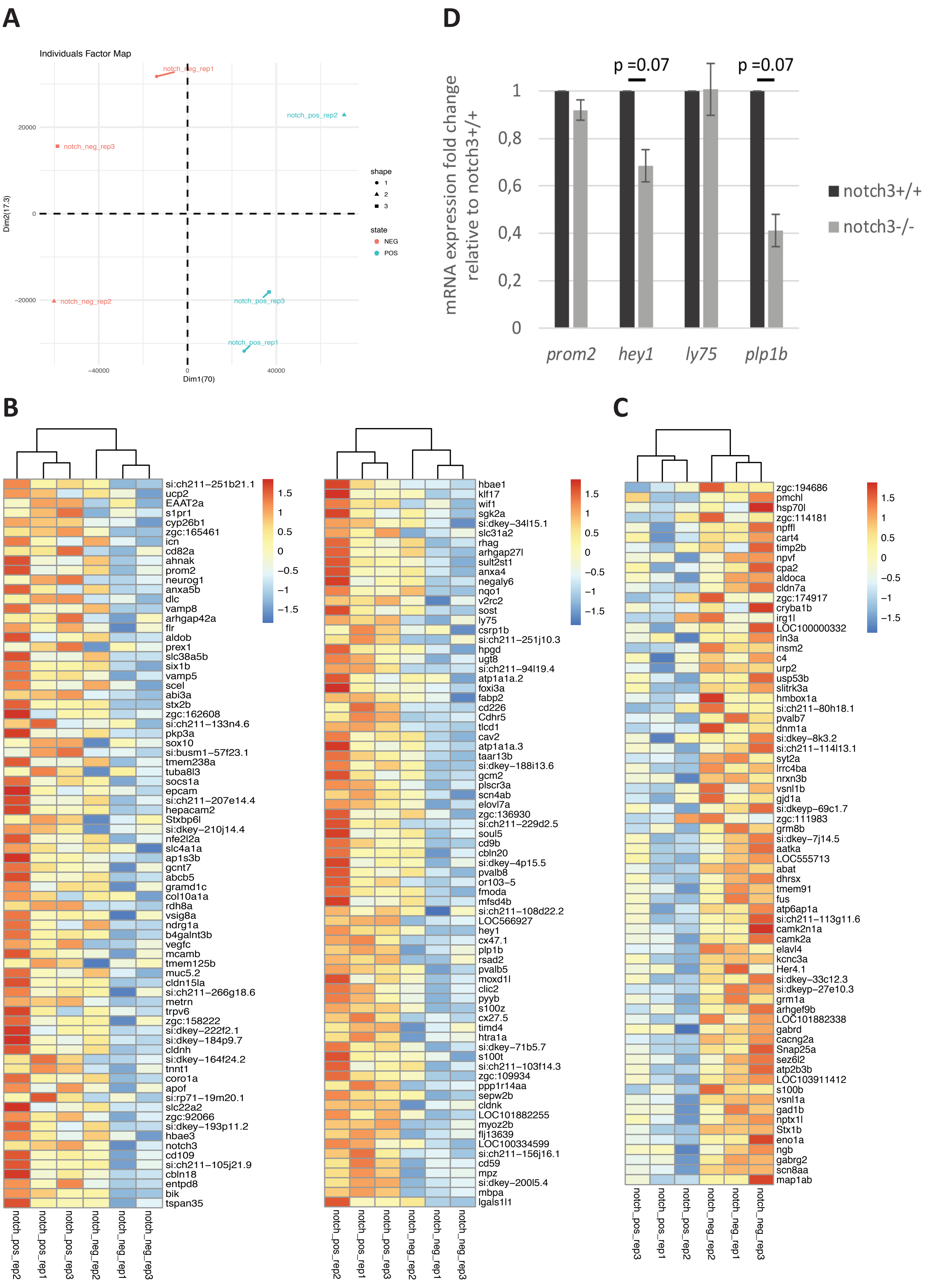

Than-Trong et al., Figure S3

Development • Supplementary information

__Figure S3__ (corresponding to Fig.2). **A-C. Validations of the differential transcriptome profiling analysis of *notch3*$^{-/-}$ and *notch3*$^{+/+}$ 7dpf RG. A.** PCA analysis on the 500 genes with the most variable expression in the different FACS sorted biological replicates (red: *notch3*$^{-/-}$; blue: *notch3*$^{+/+}$). **B.** Heat maps of the genes downregulated (**B**) and upregulated (**C**) in *notch3*$^{-/-}$ compared to $^{+/+}$ larvae. Cutoff on display: log(fold change) > 1. **D. RT-qPCR transcripts quantification for 4 potentially direct Notch3 targets** (Fig.2F), performed on RNA samples extracted from whole genotyped 7dpf larval heads. Down-regulated expression of *plp1b* and *hey1* in *notch3*$^{-/-}$ mutants was confirmed under these values, biological replicates were used as blocking factors and p-value were adjusted for multiple comparisons (see Statistics, Material and Methods, Main text).

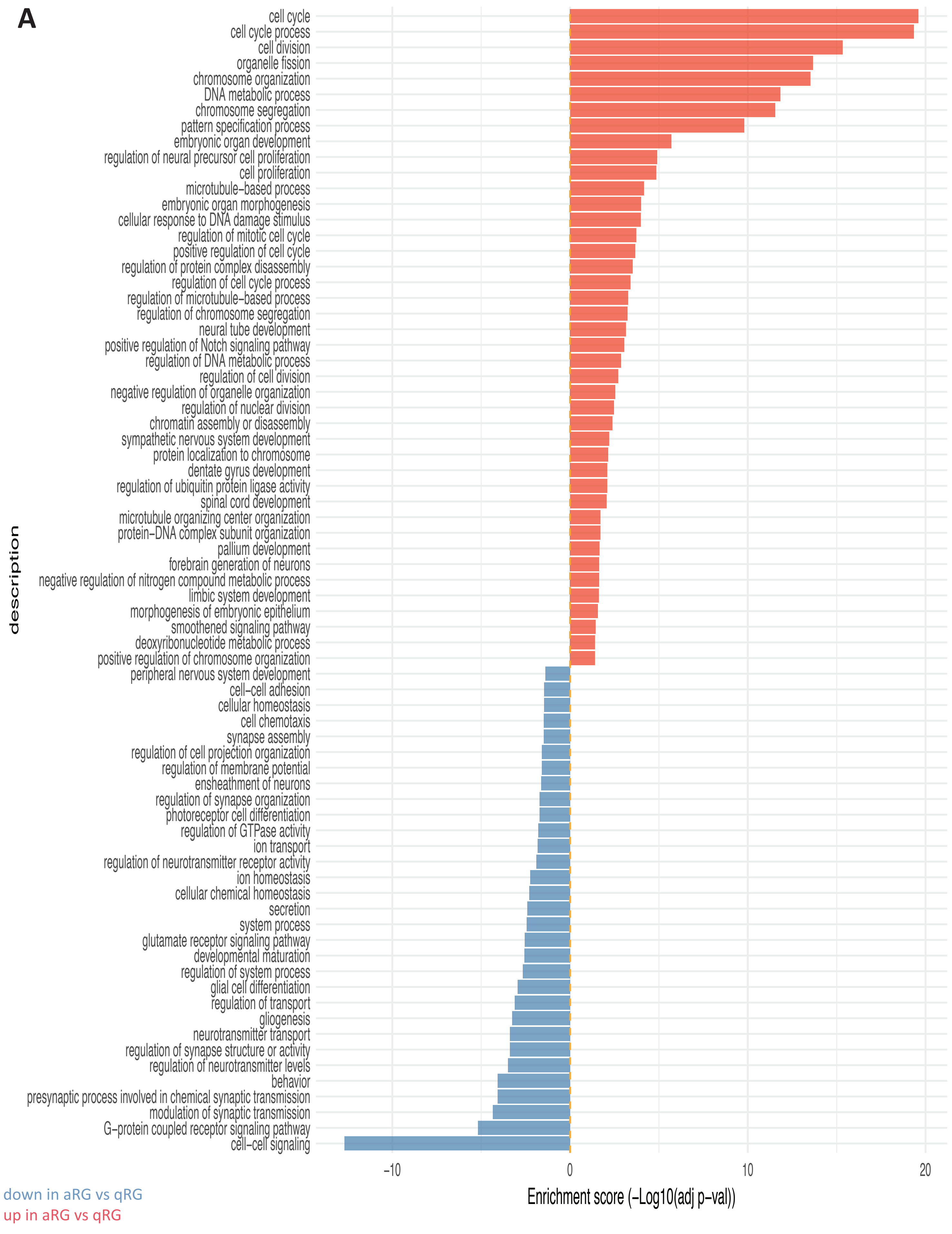

Than-Trong et al., Figure S4A

Development • Supplementary information

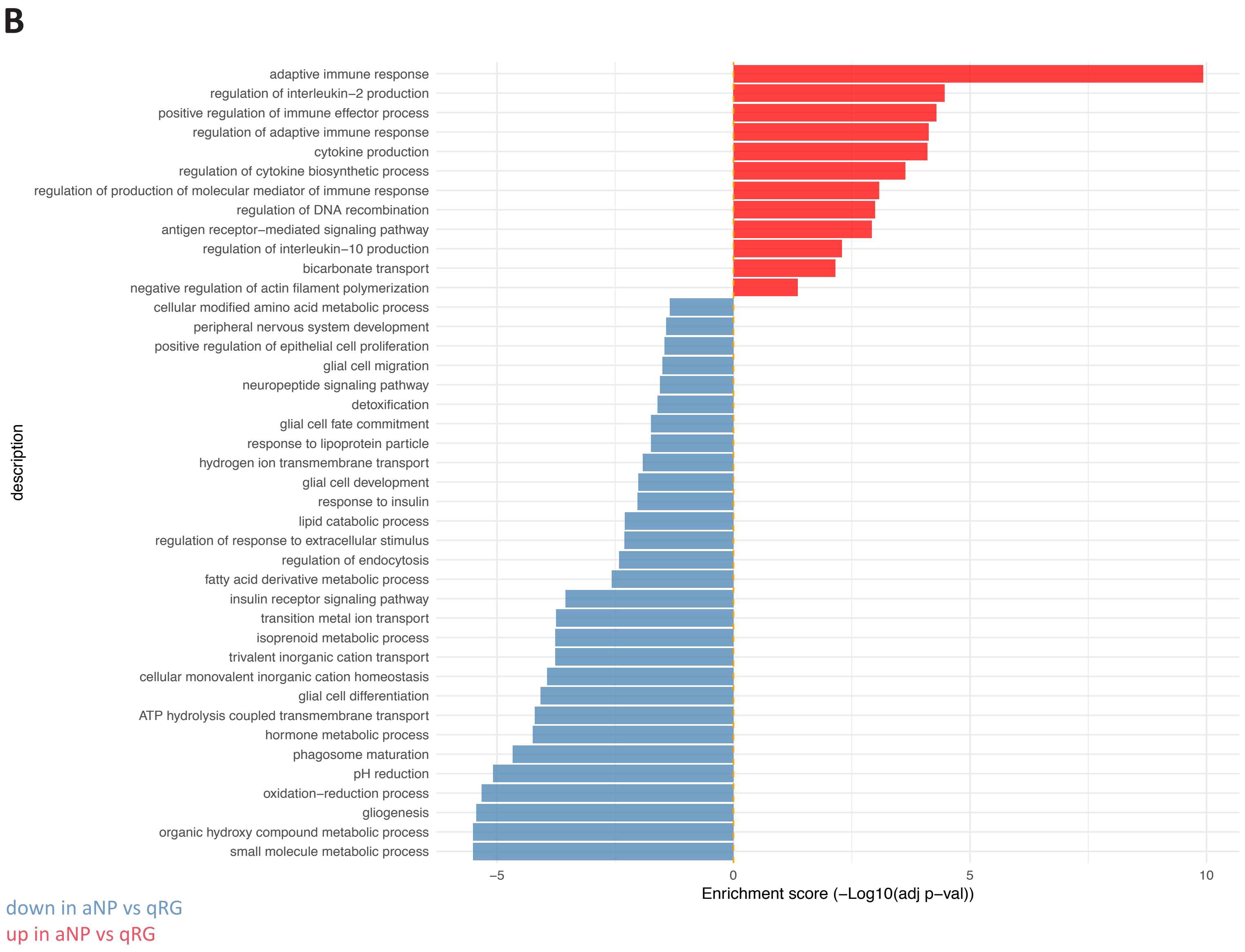

Than-Trong et al., Figure S4B

Development • Supplementary information

C

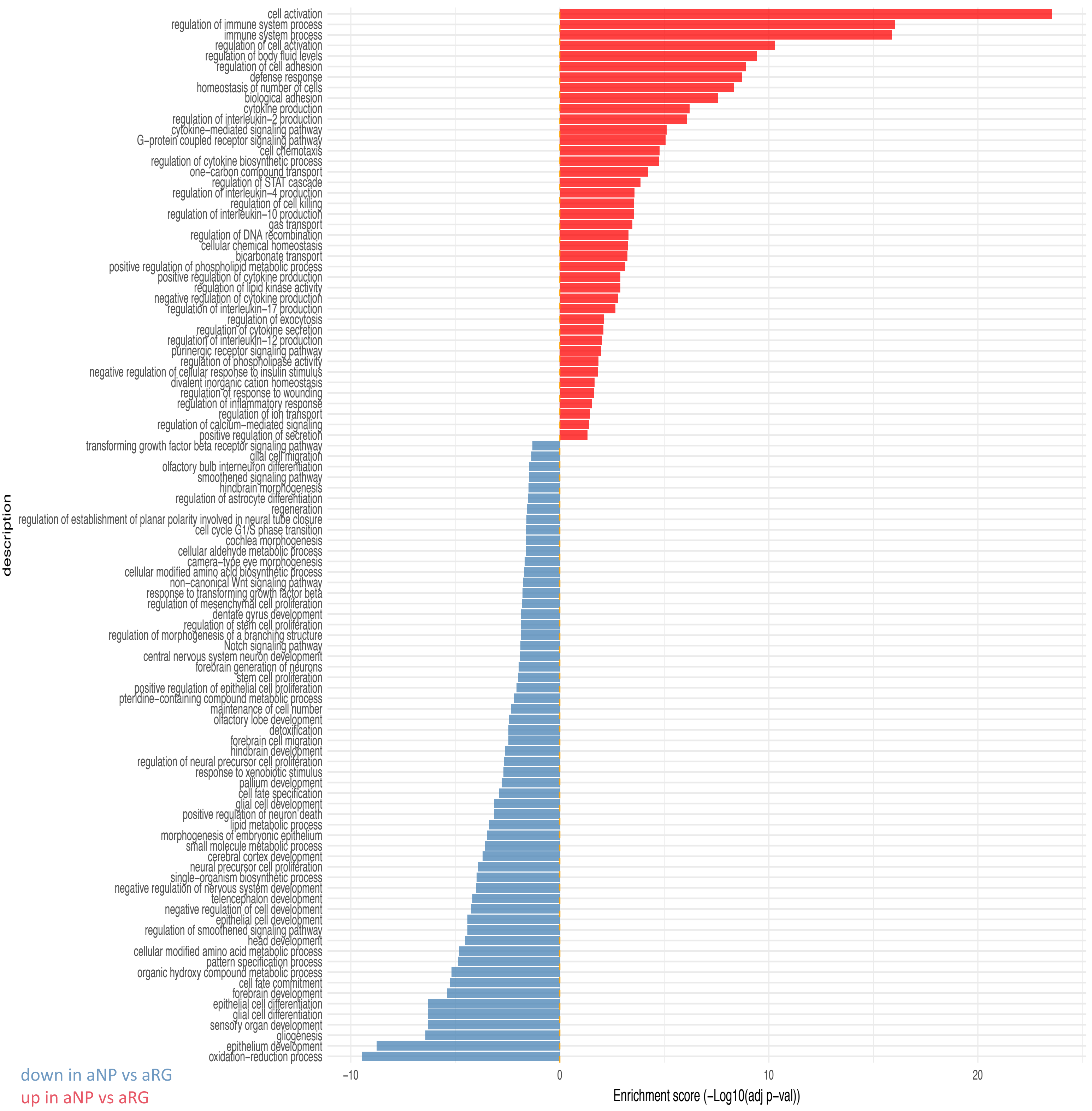

Than-Trong et al., Figure S4C

Development • Supplementary information

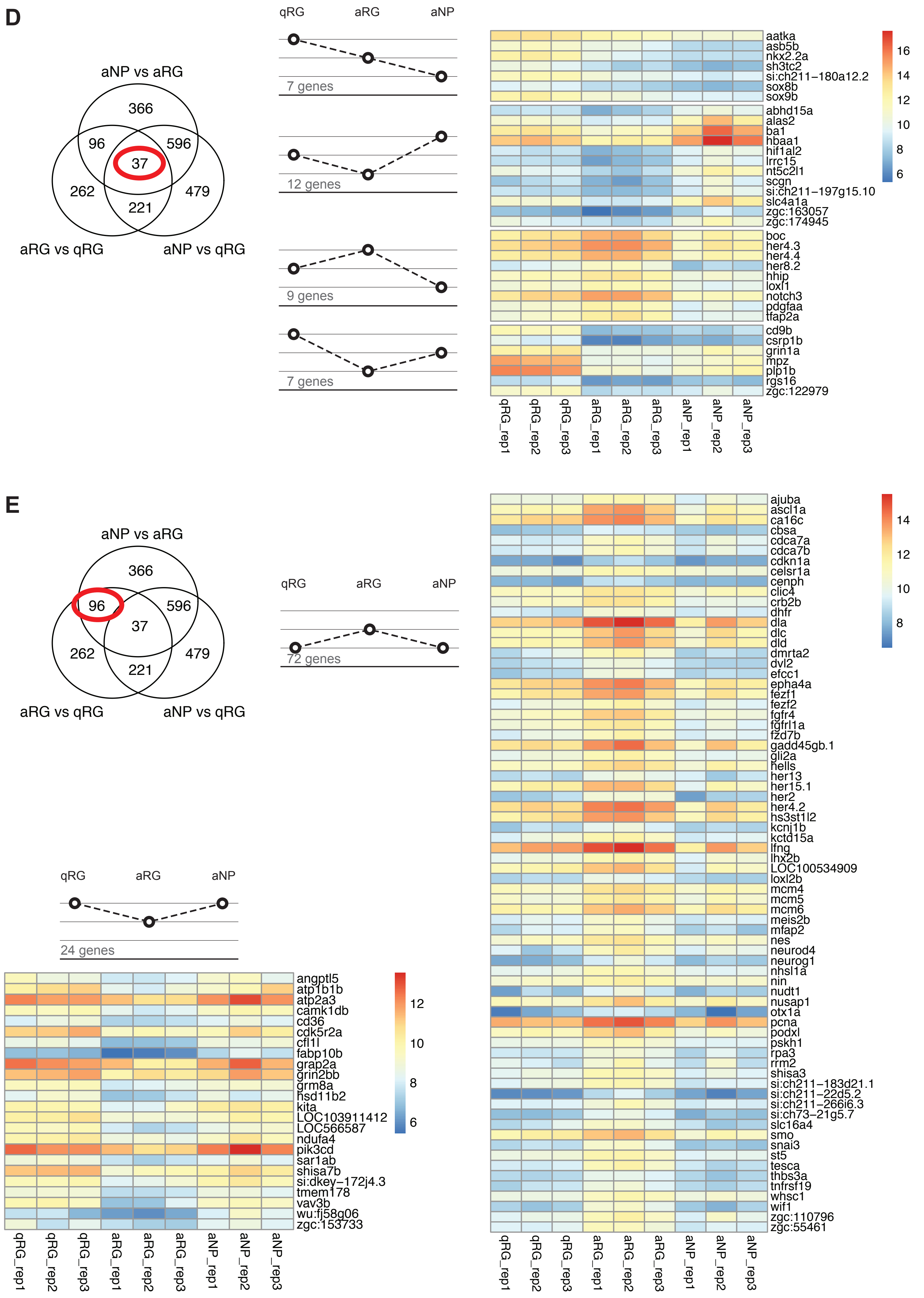

Than-Trong et al., Figure S4

Development • Supplementary information

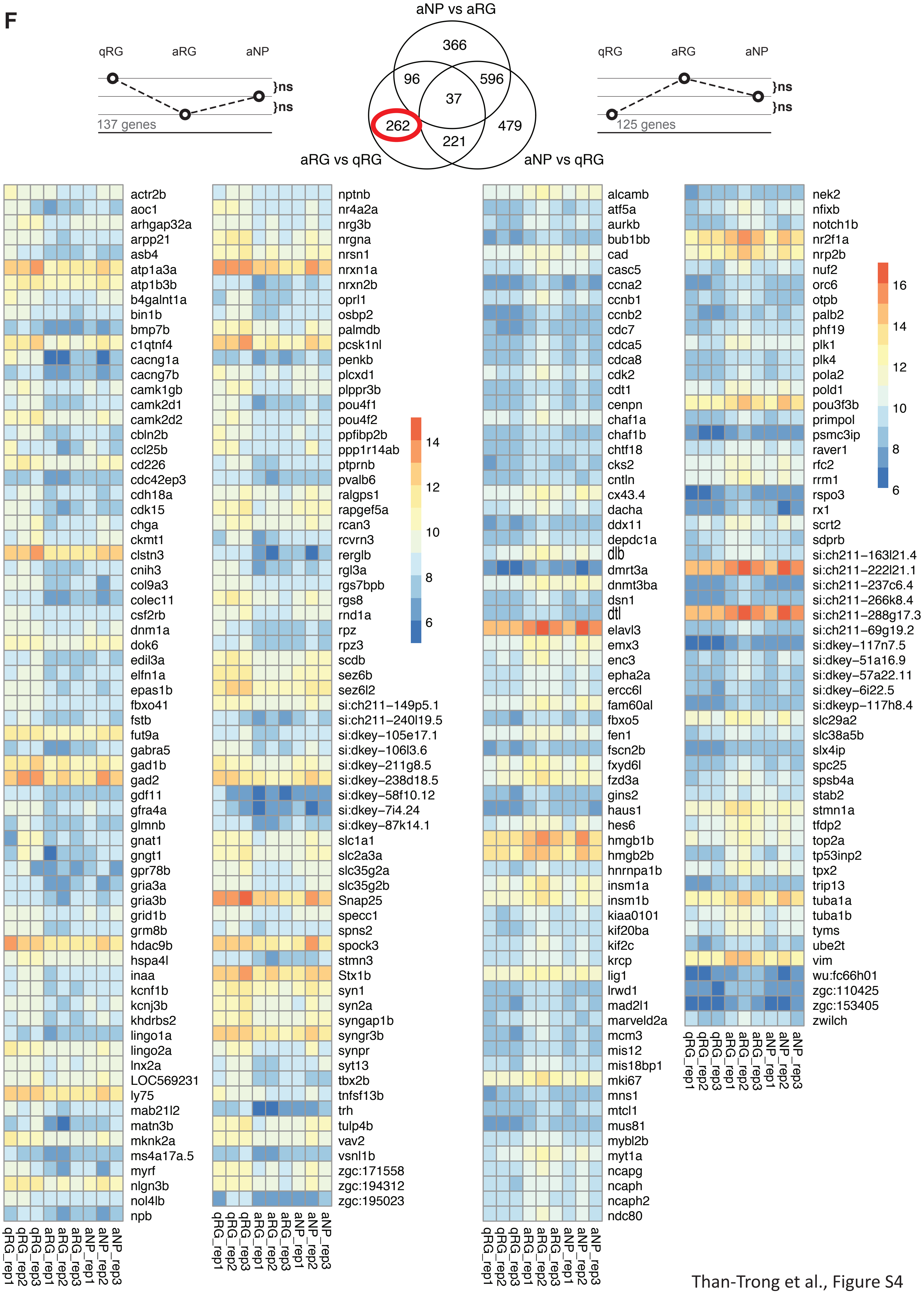

Than-Trong et al., Figure S4

Development • Supplementary information

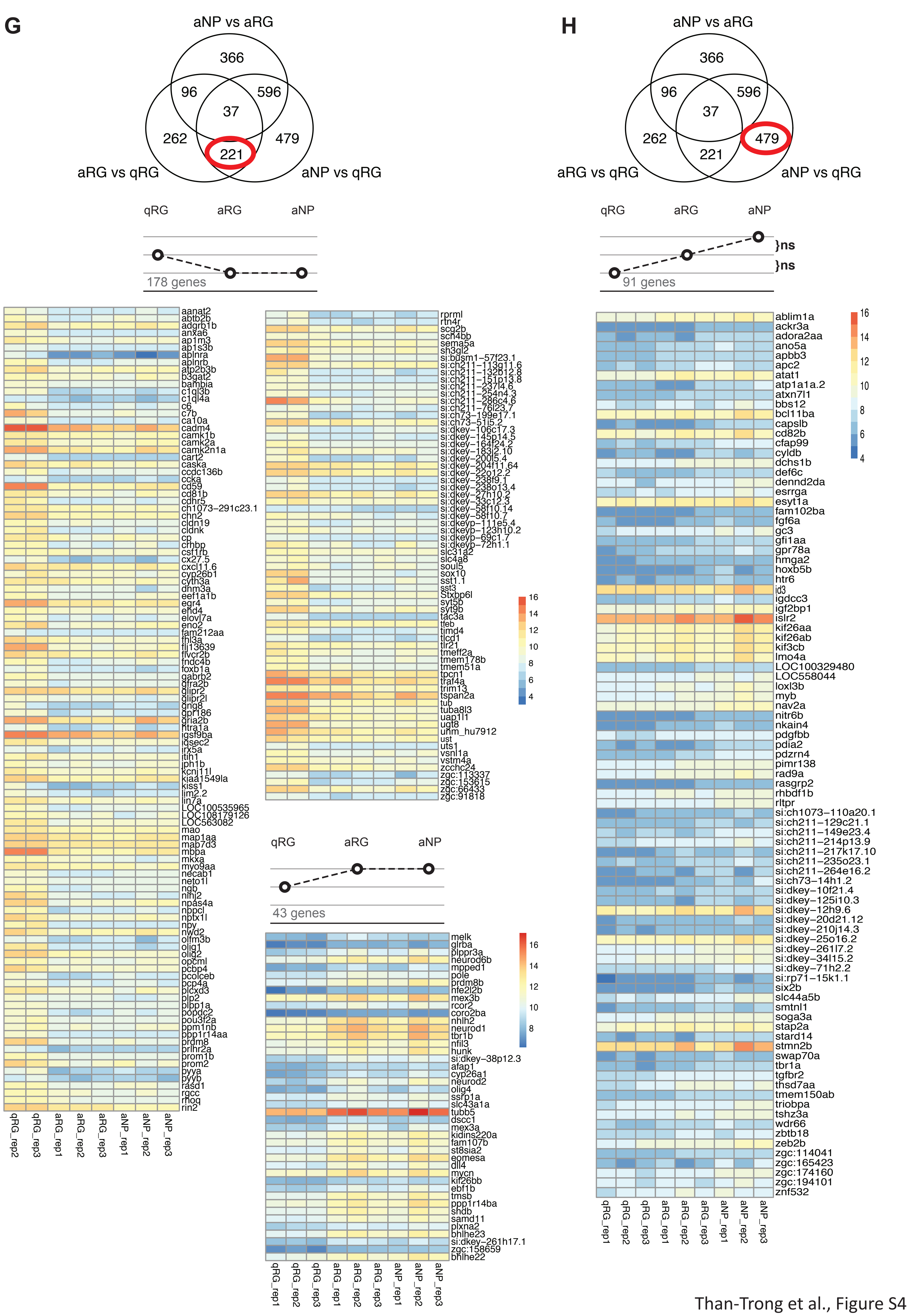

Than-Trong et al., Figure S4

Development • Supplementary information

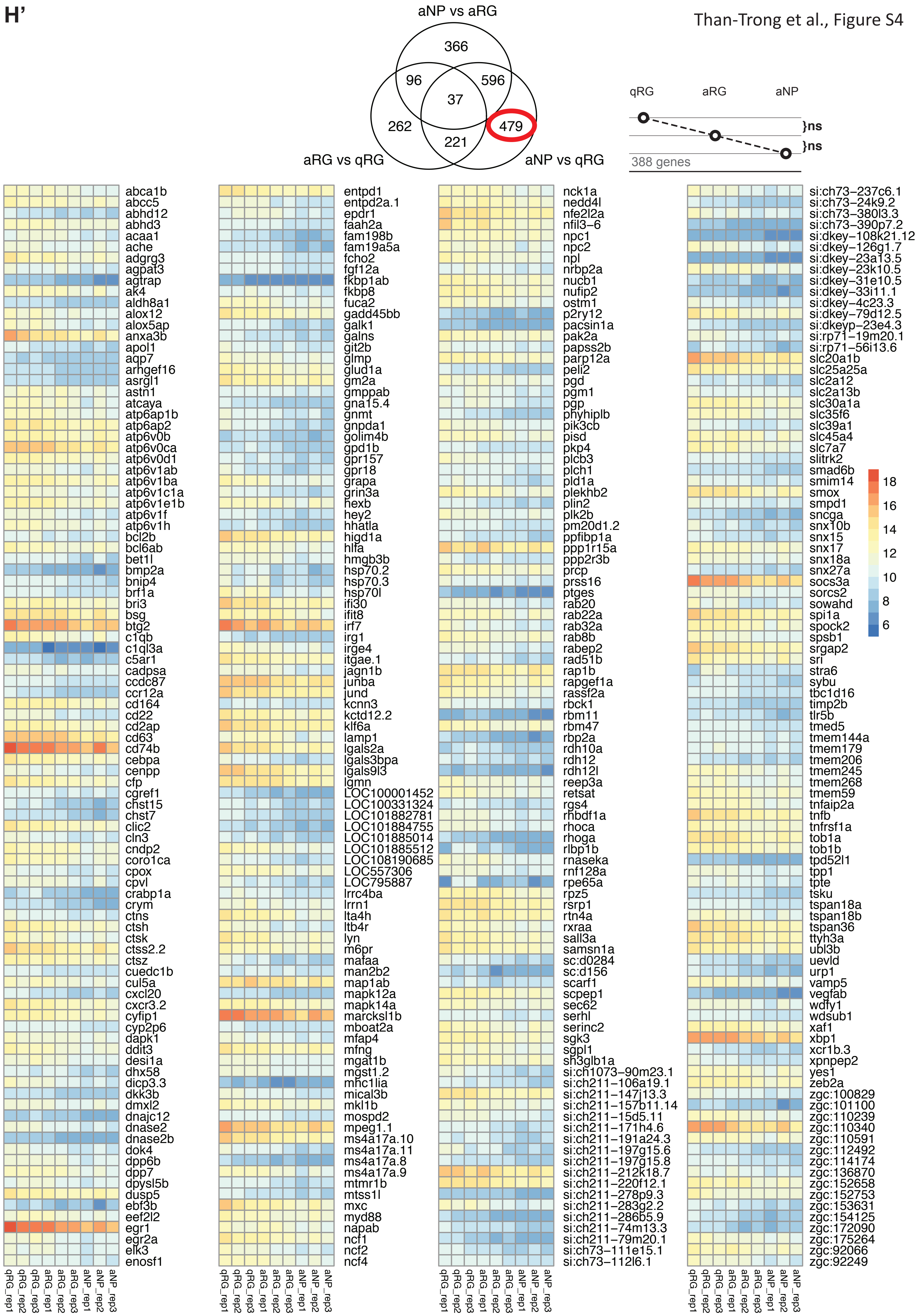

Development • Supplementary information

I

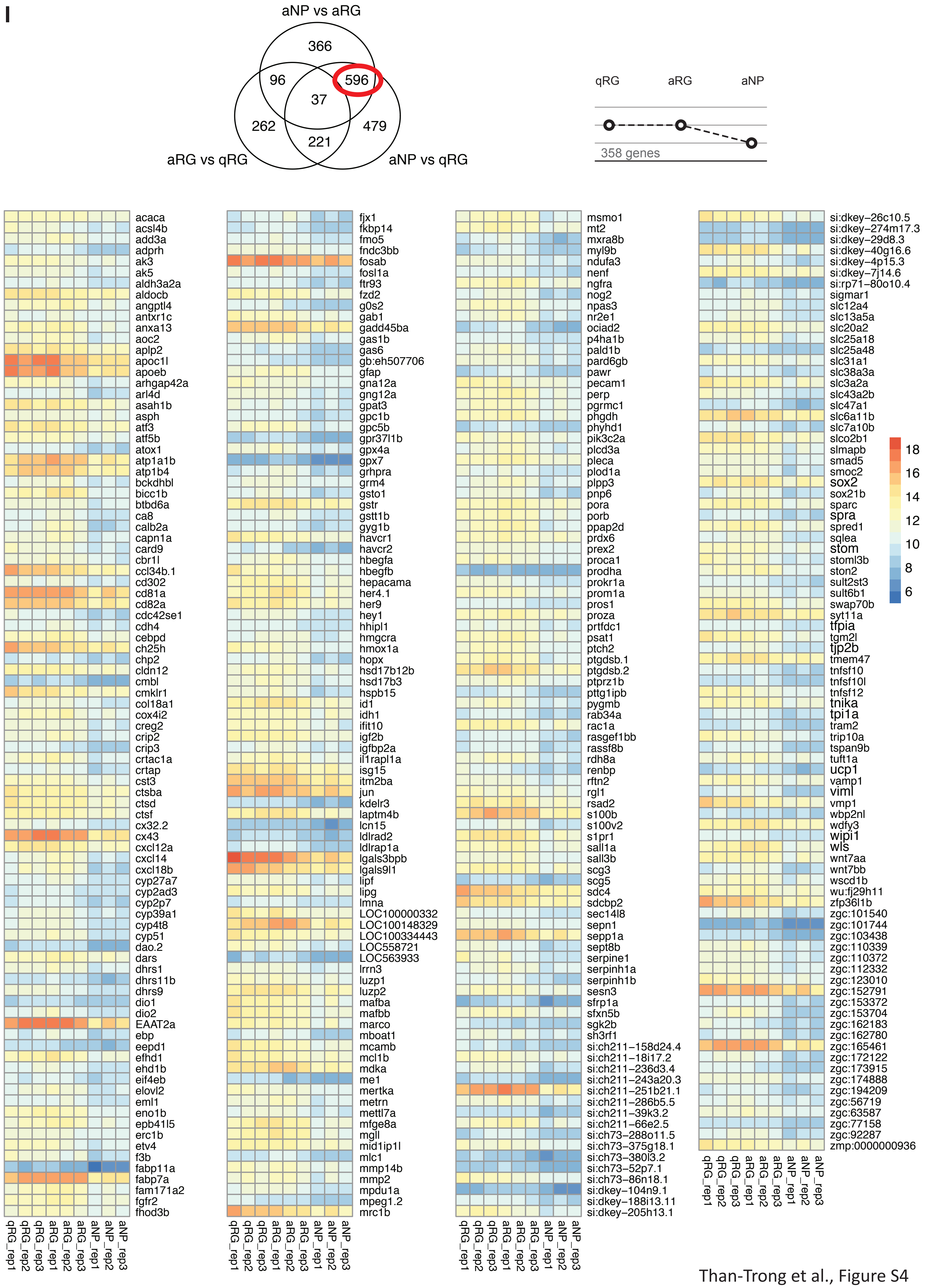

Than-Trong et al., Figure S4

Development • Supplementary information

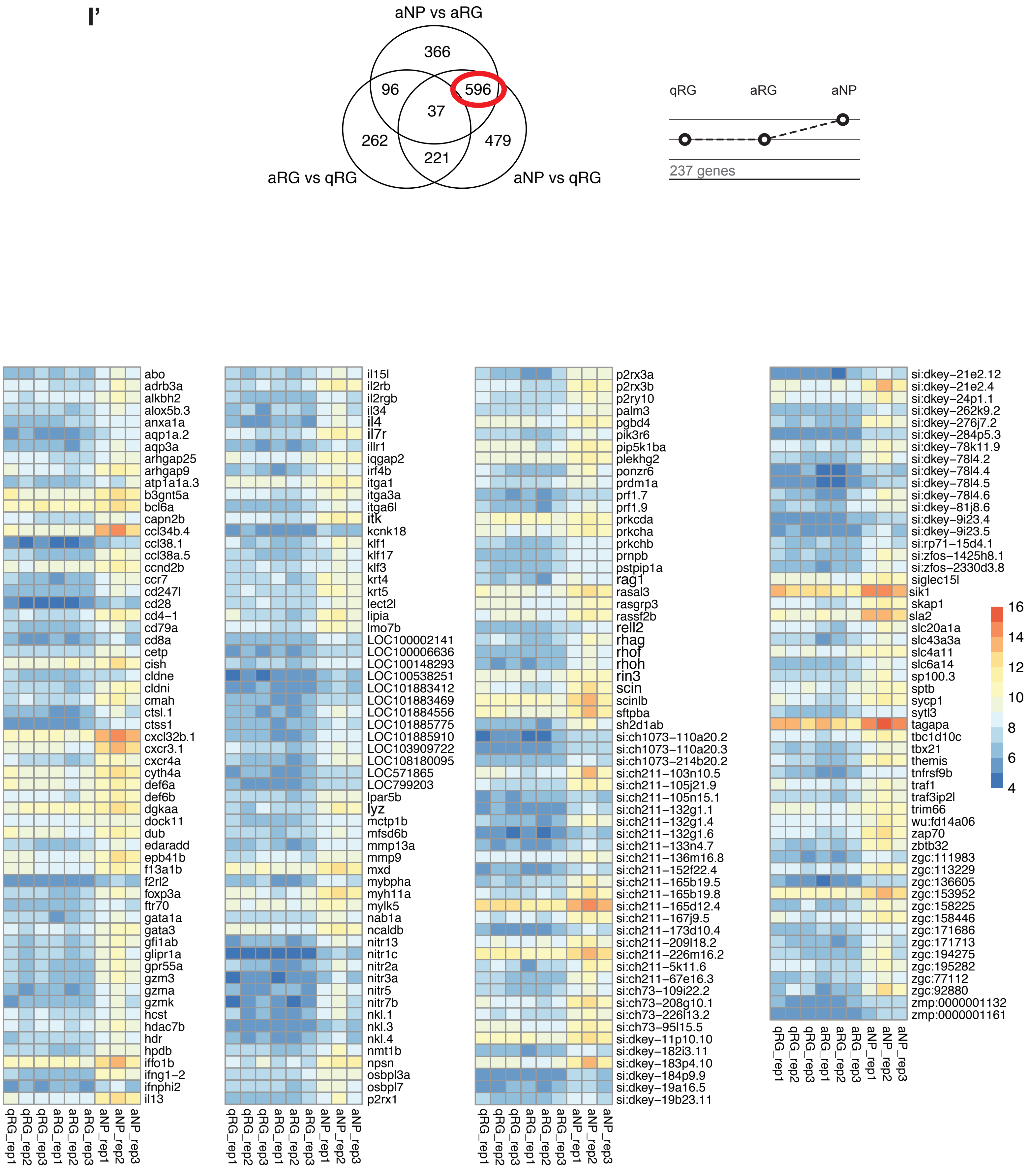

Than-Trong et al., Figure S4

Development • Supplementary information

<u>**Figure S4**</u> (related to Figure 3). **GO term and heat map analysis of the differentially expressed gene sets between qRG, aRG and aNP. A-C.** GO term analyses of DEGs between the three different progenitor cell states (**A**: qRG versus aRG, **B**: aRG versus aNP, **C**: qRG versus aNP). **D-I'.** Heat maps highlighting expression levels of the DEGs in each intersectional subcategory (venn diagrams: as in Figure 3F, with relevant category surrounded in red). In each case, a scheme depicting the variations in gene expression levels between qRG, aRG and aNP for each subcategory is also represented, with an indication of the number of genes concerned. The dotted lines linking gene expression levels between cell states indicate the most likely lineage progression. Abbreviations: ns: non-significant; rep: biological replicate.

Development • Supplementary information

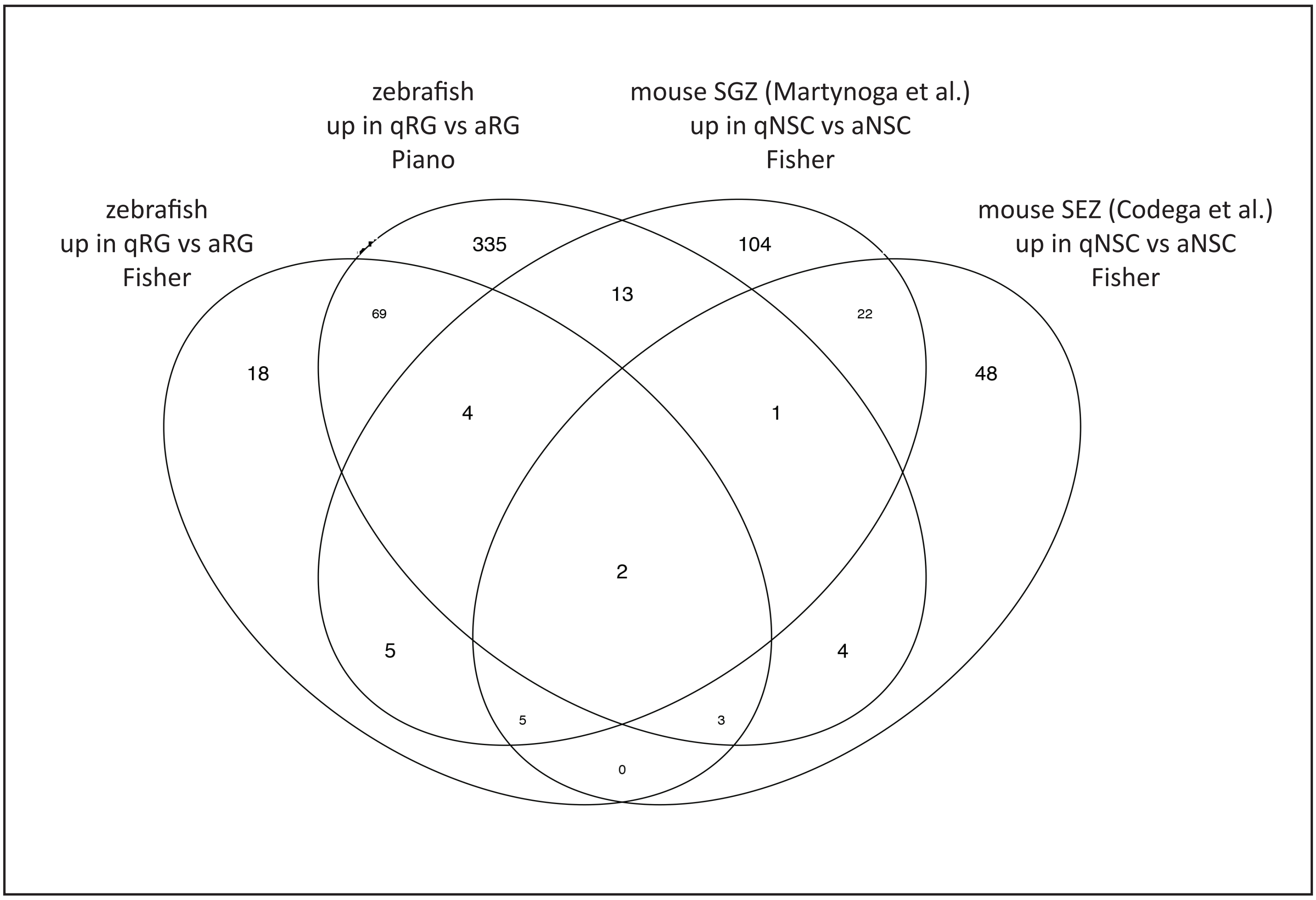

Than-Trong et al., Figure S5

Figure S5 (related to Figure 3). **Venn diagram comparing the GO terms** (Biological Process) **enriched in qNSCs versus aNSCs, statistically analyzed by over representation analysis (ORA, using a one tailed Fisher's exact test) or a functional scoring method (FSC, using runGSEA function in Piano R package), in this study and the two mouse studies Martynoga et al.** [7] **and Codega et al.** [8]**.** From Tables S11 and S12.

Development • Supplementary information

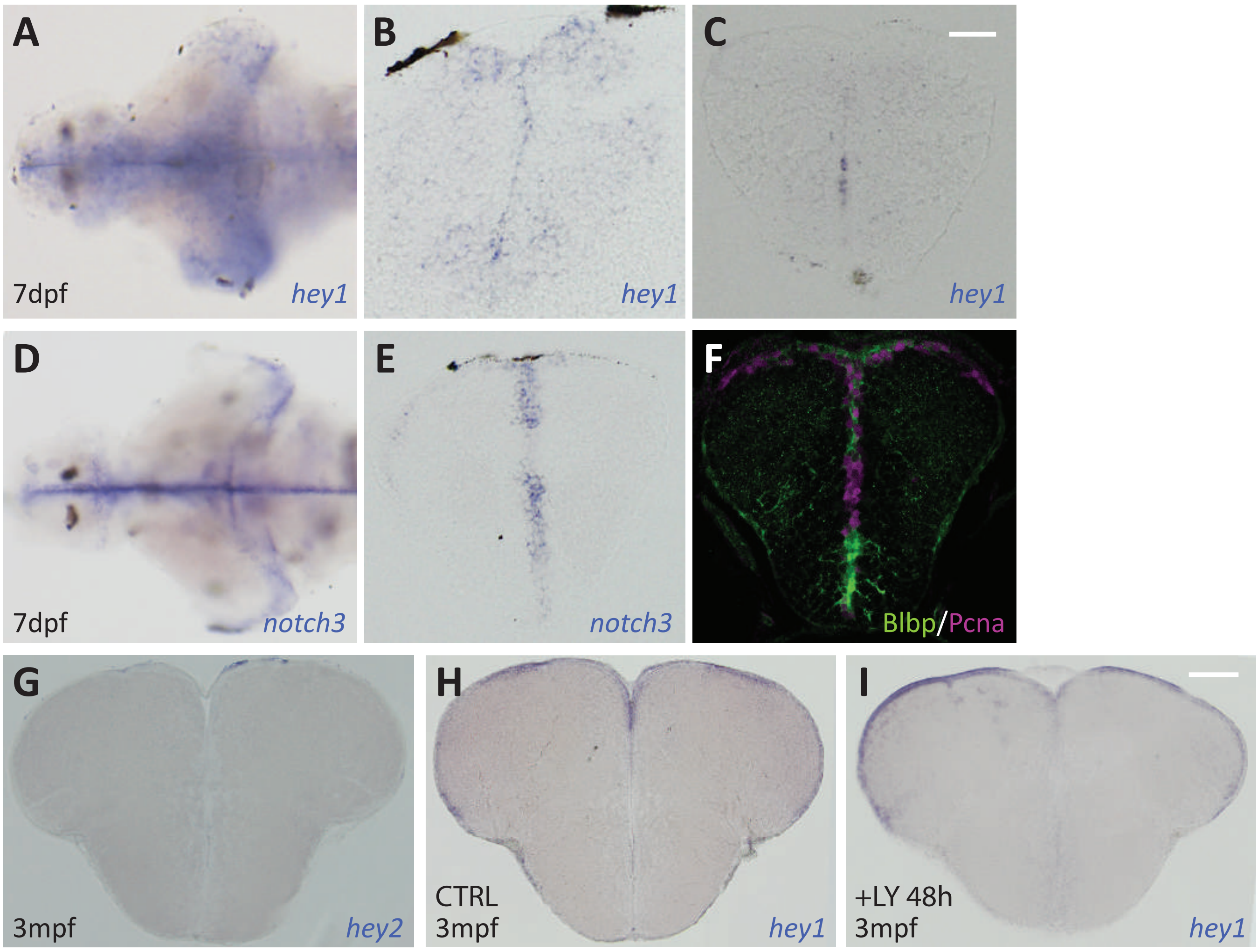

Than-Trong et al., Figure S6

__Figure S6__ (related to Figure 5). **Expression of *hey* family genes and *notch3* in the larval and adult pallium. A,B.** Expression of *hey1* revealed by *in situ* hybridization (ISH) on a whole-mount brain (**A**) and pallial cross-section (**B**) in a wildtype 7dpf larva. **C.** Expression of *hey1* revealed by ISH in a pallial cross-section of a 7dpf *notch3*$^{-/-}$ larva. Note the strong decrease compared to the wildtype sibling (**B**). **D,E.** Expression of *notch3* revealed by in situ hybridization (ISH) on a whole-mount brain (**D**) and pallial cross-section (**E**) in a wildtype 7dpf larva. **F.** Pallial cross-section at equivalent levels double immunostained for BLBP (green RG cells) and PCNA (magenta, proliferating cells). Note overall that expression of *hey1* and *notch3* are prominent at the VZ. **G.** Expression of *hey2* revealed by ISH in an adult pallial cross-section. No expression was detected (compared with *hey1* on panel H, identical to Fig.5B). **H,I.** Expression of *hey1* revealed by ISH in adult pallial cross-sections from a wildtype (H) and LY-treated (I) animals. Note that *hey1* expression is not noticeably affected by a 48-hour LY treatment Scale bars: A-F: 20µm, G-I: 50µm.

Development • Supplementary information

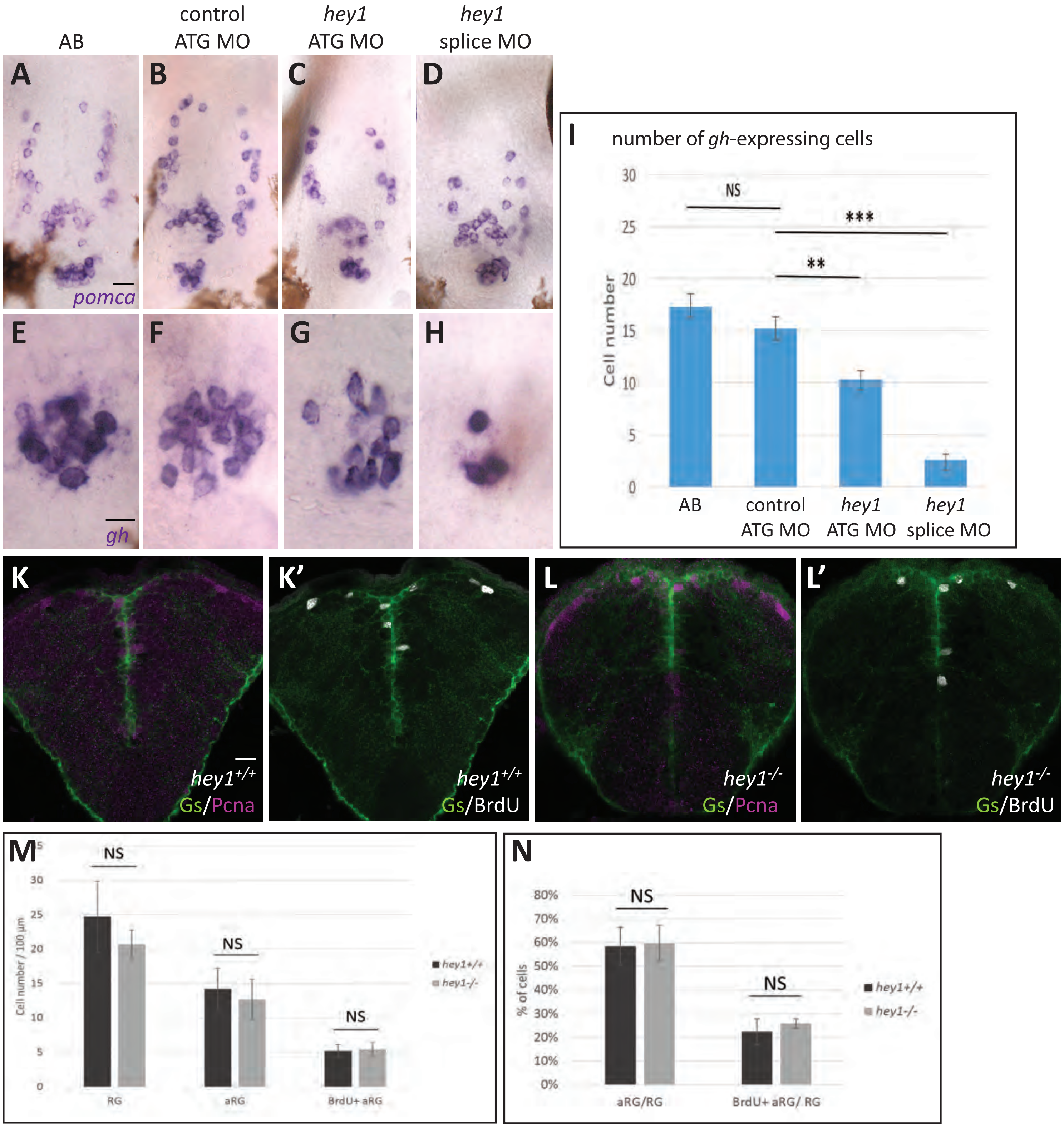

Than-Trong et al., Figure S7

<u>Figure S7</u> (related to Figure 5). **A-H. Validation of the efficiency and specificity of the *hey1* morpholinos.** Whole-mount in situ hybridization for the expression of *pomca* (A-D) and *gh* (E-H) in 72hpf larvae, wild-type (A,E) or injected at the one-cell stage with the MOs indicated (B-D, F-H). High magnification of the pituitary area. **I.** Quantification of the number of *GH*-positive neurons in embryos as in panels E-H. n=7 embryo for each condition. **: p=0.006; ***: p<0.0001. **J-N. RG proliferation is unaffected in *hey1*$^{-/-}$ larvae.** Cross-sections of the pallium in wildtype (K,K') and *hey1*$^{-/-}$ (L,L') 7dpf larvae, immunoprocessed for Gs and Pcna (K,L) or Gs and BrdU (K',L') following a short BrdU pulse, and corresponding quantification of the proportion of aRG (M) or BrdU-positive aRG (N) among the whole RG population. n=4 larvae and 250 RG cells counted for each genotype. There was no Cas3-positive cell among RG cells. Scale bars: A-D: 20µm, E-H: 10 µm, K-L': 20 µm.

Development • Supplementary information

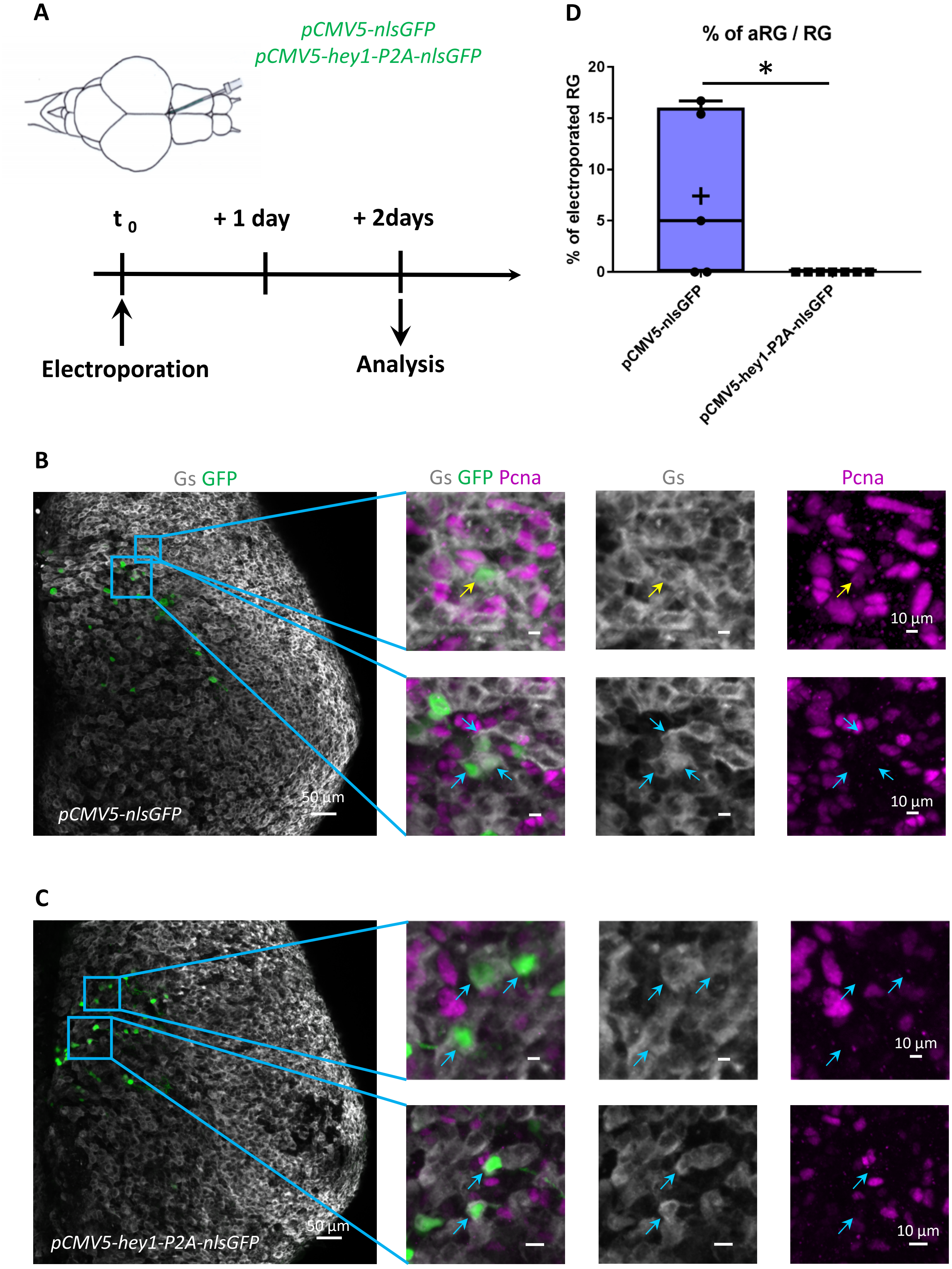

Than-Trong et al., Figure S8

Development • Supplementary information

Figure S8 (related to Figure 5). **Overexpression of *hey1* decreases proliferation in adult RG.** Electroporation of *pCMV5:nlsgfp* (A,B) or *pCMV5:hey1-P2A-nlsgfp* (A,C) in the adult ventricular zone and quantification of the proportion of PCNA-positive RG among GFP-positive RG at 2 days post-electroporation (D). B,C: whole-mount hemispheres processed for triple immunocytochemistry (GS: gray, PCNA: magenta, GFP: green), with high magnifications of the boxed areas. n=5 brains for *pCMV5:nlsgfp* and n=7 brains for *pCMV5:hey1-P2A-nlsgfp.* 63 and 105 cells were counted respectively in each condition. p=0.033, Mann-Whitney test.

hey1 wildtype sequence

```
genomic
ATGAAGAGAAATCACGATTTCAGCTCGTCGGACAGTGAGCTCGATGAGAATATCGAAGTGGAGAAGGAGAGTGCG
GATGAAAATGCCGGTGCGAATTCTCCACTCGGGTCAATGTCTCCATCCACAACCTCTCAAGTACAAGCAAGAAAA
CGTCGCAGAGGGATCATCGAGAAGCGCCGGAGGGACCGGATAAATAACAGTTTATCTGAGCTGCGCAGGCTGGTG
CCCAGCGCCTTTGAGAAACAGGGCTCAGCTAAACTAGAAAAAGCAGAAATTCTGCAGATGACCGTAGATCATTTA
AAGATGCTTCATGCTGCAGGAGGAAAAGGTTACTTTGATGCTCACGCTCTGGCCATGGATTACCGAGGACTGGGT
TTCCGCGAATGTCTAGCAGAGACTGCACGTTACCTCAGTATCATCGAGGGCCTGGACAACACAGATCCCCTCCGC
ATCCGTCTGGTTTCACATCTCAATAGCTACGCCTCTCAGAGAGAAGCTCACTCGGGTTTGGGCCACTTGGCATGG
GGTTCTGCATTTGGAACGCCTCCCAGTCACCTGGCCCACCACCTCCTCCTGCAACAGCAGCAGCAGCAGGGGGCG
CCACTGGCGCGCAGTACCAGCAGTCCTCCATCCTCAAACTCATCATCGCCCTCCTCCTCGTCTCCTTCCGCCCCG
TCAACAGAGCCCAGGTTGAGCGGGACGGTGATCAGTGAGGCAGGGCAGACGGGACCGCTAAGGGTGCCACCCAGT
ACCTCCCTTCCCCCAGGCCTCACTCCACCTACTGCATCTAAGCTTTCTCCGCCCCTCCTGACGTCACTTTCAAGC
CTGTCAGCGTTTCCCTTCCCACTGAGTGCTTTTCCTCTGCTGTCCCCAAGCTCACTGGGCCCCGCAACGCCCTCC
AGCAGCCTAGGGAAGCCCTACAGGCCCTGGAGCATGGAAATAGGGGCCTTCTGA

protein
MKRNHDFSSSDSELDENIEVEKESADENAGANSPLGSMSPSTTSQVQARKRRRGIIEKRRRDRINNSLSELRRLV
PSAFEKQGSAKLEKAEILQMTVDHLKMLHAAGGKGYFDAHALAMDYRGLGFRECLAETARYLSIIEGLDNTDPLR
IRLVSHLNSYASQREAHSGLGHLAWGSAFGTPPSHLAHHLLLQQQQQGAPLARSTSSPPSSNSSSPSSSSPSAP
STEPRLSGTVISEAGQTGPLRVPPSTSLPPGLTPPTASKLSPPLLTSLSSLSAFPFPLSAFPLLSPSSLGPATPS
SSLGKPYRPWSMEIGAF
```

hey1 mutant sequence (11bp deletion)

```
genomic
ATGAAGAGAAATCACGATGGACAGTGAGCTCGATGAGAATATCGAAGTGGAGAAGGAGAGTGCGGATGAAAATGC
CGGTGCGAATTCTCCACTCGGGTCAATGTCTCCATCCACAACCTCTCAAGTACAAGCAAGAAAACGTCGCAGAGG
GATCATCGAGAAGCGCCGGAGGGACCGGATAAATAACAGTTTATCTGAGCTGCGCAGGCTGGTGCCCAGCGCCTT
TGAGAAACAGGGCTCAGCTAAACTAGAAAAAGCAGAAATTCTGCAGATGACCGTAGATCATTTAAAGATGCTTCA
TGCTGCAGGAGGAAAAGGTTACTTTGATGCTCACGCTCTGGCCATGGATTACCGAGGACTGGGTTTCCGCGAATG
TCTAGCAGAGACTGCACGTTACCTCAGTATCATCGAGGGCCTGGACAACACAGATCCCCTCCGCATCCGTCTGGT
TTCACATCTCAATAGCTACGCCTCTCAGAGAGAAGCTCACTCGGGTTTGGGCCACTTGGCATGGGGTTCTGCATT
TGGAACGCCTCCCAGTCACCTGGCCCACCACCTCCTCCTGCAACAGCAGCAGCAGCAGGGGGCGCCACTGGCGCG
CAGTACCAGCAGTCCTCCATCCTCAAACTCATCATCGCCCTCCTCCTCGTCTCCTTCCGCCCCGTCAACAGAGCC
CAGGTTGAGCGGGACGGTGATCAGTGAGGCAGGGCAGACGGGACCGCTAAGGGTGCCACCCAGTACCTCCCTTCC
CCCAGGCCTCACTCCACCTACTGCATCTAAGCTTTCTCCGCCCCTCCTGACGTCACTTTCAAGCCTGTCAGCGTT
TCCCTTCCCACTGAGTGCTTTTCCTCTGCTGTCCCCAAGCTCACTGGGCCCCGCAACGCCCTCCAGCAGCCTAGG
GAAGCCCTACAGGCCCTGGAGCATGGAAATAGGGGCCTTCTGA

protein
MKRNHDGQ*AR*EYRSGEGECG*KCRCEFSTRVNVSIHNLSSTSKKTSQRDHREAPEGPDK*QFI*AAQAGAQRL
*ETGLS*TRKSRNSADDRRSFKDASCCRRKRLL*CSRSGHGLPRTGFPRMSSRDCTLPQYHRGPGQHRSPPHPSG
FTSQ*LRLSERSSLGFGPLGMGFCIWNASQSPGPPPPPATAAAAGGATGAQYQQSSILKLIIALLLVSFRPVNRA
QVERDGDQ*GRADGTAKGATQYLPSPRPHSTYCI*AFSAPPDVTFKPVSVSLPTECFSSAVPKLTGPRNALQQPR
EALQALEHGNRGLLX
```

Wildtype Hey1 protein at deletion site

```
atgaagagaaatcacgatttcagctcgtcggacagt
 M  K  R  N  H  D  F  S  S  S  D  S
```

Hey1 mutant (11bp deletions) protein at deletion site

```
atgaagagaaatcacgat-----------ggacagt
 M  K  R  N  H  D              G  Q
```

Than-Trong et al., Figure S9

__Figure S9__ (related to Figure 5). hey1 genomic DNA sequences and predicted amino acid sequences in wildtype and in the novel *hey1* mutant used in this study. Sequences were obtained from individual embryos.

Development • Supplementary information

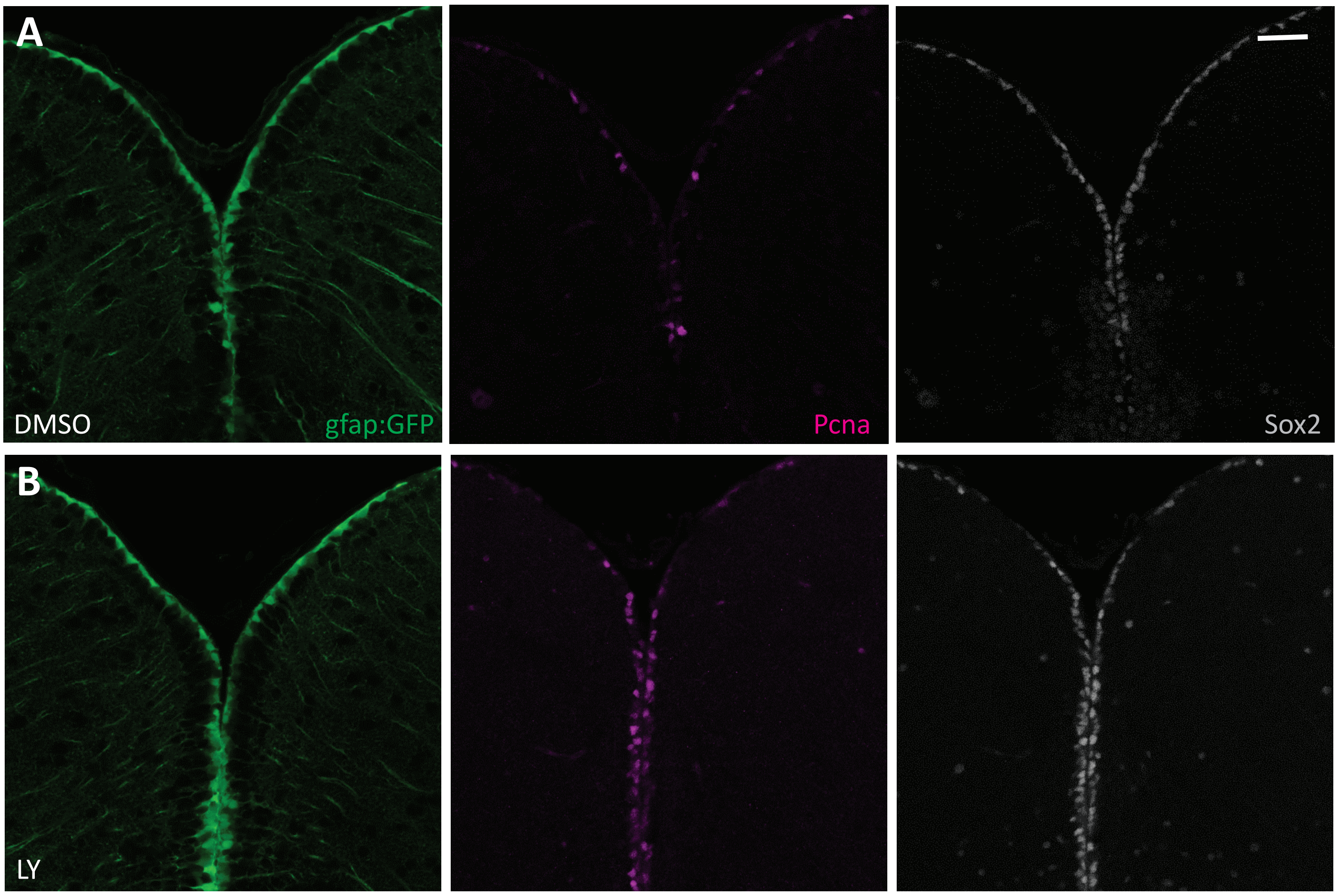

Than-Trong et al., Figure S10

Figure S10 (related to Figure 6). Notch blockade reactivates quiescent RG and does not affect their expression of Sox2. Cross sections of the adult pallium in *gfap:gfp* transgenic animals following 48 hours of DMSO (**A**) or LY411575 (**B**) treatment, triple immunostained for GFP (green), PCNA (magenta) and Sox2 (grey). Scale bars: 20µm.

Development • Supplementary information
